# *SPL33*, encoding an eEF1A-like protein, negatively regulates cell death and defense responses in rice

**DOI:** 10.1093/jxb/erx001

**Published:** 2017-02-11

**Authors:** Shuai Wang, Cailin Lei, Jiulin Wang, Jian Ma, Sha Tang, Chunlian Wang, Kaijun Zhao, Peng Tian, Huan Zhang, Changyan Qi, Zhijun Cheng, Xin Zhang, Xiuping Guo, Linglong Liu, Chuanyin Wu, Jianmin Wan

**Affiliations:** 1 Institute of Crop Science, Chinese Academy of Agriculture Sciences/National Key Facility for Crop Gene Resources and Genetic Improvement, Beijing 100081, China; 2 Key Laboratory of Crop Genetics and Germplasm Enhancement/Jiangsu Provincial Center of Plant Gene Engineering, Nanjing Agricultural University, Nanjing 210095, China

**Keywords:** Defense responses, eukaryotic translation elongation factor 1 alpha (eEF1A), lesion-mimic mutant, *Oryza sativa*, programed cell death, *SPL33.*

## Abstract

Lesion-mimic mutants are useful to dissect programmed cell death and defense-related pathways in plants. Here we identified a new rice lesion-mimic mutant, *spotted leaf 33* (*spl33*) and cloned the causal gene by a map-based cloning strategy. *SPL33* encodes a eukaryotic translation elongation factor 1 alpha (eEF1A)-like protein consisting of a non-functional zinc finger domain and three functional EF-Tu domains. *spl33* exhibited programmed cell death-mediated cell death and early leaf senescence, as evidenced by analyses of four histochemical markers, namely H_2_O_2_ accumulation, cell death, callose accumulation and TUNEL-positive nuclei, and by four indicators, namely loss of chlorophyll, breakdown of chloroplasts, down-regulation of photosynthesis-related genes, and up-regulation of senescence-associated genes. Defense responses were induced in the *spl33* mutant, as shown by enhanced resistance to both the fungal pathogen *Magnaporthe oryzae* and the bacterial pathogen *Xanthomonas oryzae* pv. *oryzae* and by up-regulation of defense response genes. Transcriptome analysis of the *spl33* mutant and its wild type provided further evidence for the biological effects of loss of *SPL33* function in cell death, leaf senescence and defense responses in rice. Detailed analyses showed that reactive oxygen species accumulation may be the cause of cell death in the *spl33* mutant, whereas uncontrolled activation of multiple innate immunity-related receptor genes and signaling molecules may be responsible for the enhanced disease resistance observed in *spl33*. Thus, we have demonstrated involvement of an eEF1A-like protein in programmed cell death and provided a link to defense responses in rice.

## Highlight

Loss-of-function of the rice eEF1A-like protein SPL33 causes a lesion-mimic phenotype by triggering programmed cell death, and increases disease resistance, likely through activating defense pathways.

## Introduction

Plants have developed elaborate mechanisms of protection from attack by pathogens, the most common of which is the hypersensitive response (HR), which triggers rapid programmed cell death (PCD) to inhibit further invasion or proliferation of many pathogens in host plant tissues (Heath, 2000). Typical physiological processes associated with the HR include systemic signals such as bursts of reactive oxygen species (ROS) and production of free radicals, induction of pathogenesis-related (PR) genes, accumulation of antimicrobial compounds, and cell wall fortification through callose deposition (Qiao *et al.*, 2010; Shirsekar *et al.*, 2014). Although the HR plays an important role in host resistance to pathogens, its underlying molecular mechanisms are not fully understood.

As a means of dissecting HR-mediated PCD, researchers have identified and characterized a number of lesion-mimic mutants (LMMs) in a range of plant species, including *Arabidopsis* (Lorrain *et al.*, 2004), maize (Johal *et al.*, 1995) and rice (Shirsekar *et al.*, 2014; Fekih *et al.*, 2015; Wang *et al.*, 2015*a*). These mutants spontaneously develop localized cell death lesions resembling those caused by HR in the absence of pathogen infection, abiotic stress or mechanical damage (Xu *et al.*, 2014). Many LMMs display significantly enhanced resistance to disease (Wang *et al.*, 2015*a*; Wang *et al.*, 2015*b*) and it is believed that such mutants are ideal tools for deciphering the signal pathways of PCD and defense responses in plants. Therefore, cloning and characterization of the genes involved in LMMs should aid the elucidation of molecular mechanisms underlying plant disease resistance, possibly opening a way to development of broad-spectrum disease resistance (Wang *et al.*, 2015*b*).

Previous studies have shown that the functional alleles of LMM genes encode various types of different proteins, including membrane-associated proteins (Lorrain *et al.*, 2004; Noutoshi *et al.*, 2006), ion channel proteins (Rostoks *et al.*, 2006; Mosher *et al.*, 2010), zinc-finger proteins (Wang *et al.*, 2005), transcription factors (Yamanouchi *et al.*, 2002; Li *et al.*, 2004), E3 ubiqutin ligase (Zeng *et al.*, 2004), porphyrin (Ishikawa *et al.*, 2001), oxidoreductases (Tanaka *et al.*, 2003; Yang *et al.*, 2004), protein kinases (Liang *et al.*, 2003; Wang *et al.*, 2015*c*), clathrin-associated adaptor protein (Qiao *et al.*, 2010), nucleotide-binding site leucine-rich repeat (NBS-LRR) type proteins (Tang *et al.*, 2011), splicing factors (Chen *et al.*, 2012), UDP-*N*-acetylglucosamine pyrophosphorylase (Wang *et al.*, 2015*b*), and AAA-type ATPase (Fekih *et al.*, 2015), as well as proteins involved in biosynthesis or metabolism of fatty acids/lipids, porphyrin and phenolic compounds (Undan *et al.*, 2012). Thus the molecular mechanisms regulating lesion-mimic cell death and defense response in plants seem to be very complicated, and the study of LMMs not only identifies the mutated gene loci but also indicates how they might be involved in various physiological and defense-related signals (Undan *et al.*, 2012).

Eukaryotic translation elongation factor 1 alpha (eEF1A) has a pivotal role in protein synthesis by catalysing GTP-dependent binding of aminoacyl-tRNA to the acceptor site of the ribosome, and is the second most abundant protein in eukaryote cells after actin (Mateyak and Kinzy, 2010). Besides its canonical role in translation, eEF1A has been implicated in PCD (apoptosis) in higher vertebrates (Mateyak and Kinzy, 2010). In mammals, eEF1A has two isoforms, eEF1A1 and eEF1A2, which are encoded by different genes in the e*EF1A* gene family. eEF1A1 is expressed in all tissues and has pro-apoptotic properties and a possible anti-apoptotic behavior; eEF1A2 is present only in brain, heart, and skeletal muscle and has anti-apoptotic properties in ovarian, breast, pancreatic, liver, and lung cancer (Abbas *et al.*, 2015; Migliaccio *et al.*, 2015). It was suggested that oncogenes encoding eEF1As could be used as potential prognostic markers for certain cancers, and perhaps research on those genes could lead to identification of novel therapeutic targets (Lee, 2003). Although a handful of genes encoding eEF1As have been cloned and characterized in plants (Xu *et al.*, 2007), the biological phenotypes resulting from *eEF1A* gene mutations have not been reported, and thus the question of whether *eEF1A* genes are involved in plant PCD and defense responses remains to be answered.

In order to further elucidate the molecular mechanism of PCD and defense response in plants, we isolated and characterized a new rice lesion-mimic mutant named *spotted leaf 33* (*spl33*). This mutant exhibits spotted leaves and enhances resistance to both the rice blast and bacterial blight diseases. The *SPL33* allele was identified through a map-based cloning strategy combined with sequencing, and was predicted to encode an eEF1A-like protein. This gene is constitutively expressed in all tissues and developmental stages examined, and its encoded protein, SPL33, is localized in the endoplasmic reticulum (ER). Cell death, early leaf senescence and defense responses were observed in the *spl33* mutant. These results indicated that loss of *SPL33* function was the cause of regulated cell death, early senescence and enhanced defense responses.

## Materials and methods

### Plant materials and growth conditions

The rice spotted leaf mutant *spl33* was isolated from an ethyl methane sulfonate mutant pool of the *japonica* rice cv. Nipponbare. For light treatments the middle part of leaf blades of seedlings in a growth chamber (12 h of light at 30 °C/12 h of darkness at 20 °C) was wrapped with aluminum foil to block light entry. Reciprocal crosses were made between *spl33* and wild type (WT) for preliminary genetic analysis, and detailed genetic analysis was performed on an F_2_ population from a cross between *spl33* and *indica* cv. Dular. All F_2_ individuals and corresponding parents were grown in a paddy field at the Changping Experimental Station of the Institute of Crop Science from April to October.

### DNA extraction and molecular marker development

Genomic DNA was extracted from frozen young leaves using the cetyltrimethylammonium bromide (CTAB) method (Murray and Thompson, 1980). Insertion and deletion (InDel) markers were developed as described by Ma *et al.* (2015).

### Linkage analysis and mapping of *spl33*

Two hundred and twelve InDel markers evenly distributed across all the 12 chromosomes (Lei *et al.*, 2013) were screened for polymorphisms between the parents used. Twenty individuals with the *spl33* phenotype and 20 with WT phenotype from an F_2_ population of *spl33*/Dular were initially used for linkage analysis; 476 F_2_ individuals with the *spl33* phenotype were used for fine-mapping. InDel marker development and PCR amplifications were performed as described previously (Ma *et al.*, 2015). Markers used in this study are listed in Supplementary Table S1 at *JXB* online.

### Complementation and overexpression of *SPL33* in *spl33* mutant plants

For complementation of the *spl33* mutation, a 9881 bp WT genomic DNA fragment containing the entire *SPL33* coding region along with 2732 bp upstream and 2851 bp downstream sequences was amplified by PCR using the primers pP1305F and pP1305R (see Supplementary Table S2). The PCR product was inserted into the binary vector pCAMBIA1305.2 to generate the transformation plasmid p*GSPL33*. To construct an overexpression vector, the 1968 bp cDNA sequence of *SPL33* was amplified by PCR using the primers pC1390F and pC1390R (Supplementary Table S2), inserted downstream of the ubiquitin promoter, and then fused in the vector pCUbi1390, resulting in the plasmid p*Ubi::SPL33*. To determine the function of domains in *SPL33*, the cDNA fragments of 1–669 bp (spanning the amino acids 1–223) and 670–1968 bp (spanning the amino acids 224–655) were amplified using the primers p1390-SPL33^1–223^F/R and p1390-SPL33^224–655^F/R, respectively, inserted downstream of the ubiquitin promoter in pCUbi1390, resulting in p*Ubi::SPL33*^1–223^ and p*Ubi::SPL33*^224–655^. All the constructs were verified by sequencing and subsequently introduced into *spl33* by *Agrobacterium tumefaciens*-mediated transformation as described previously (Jeon *et al.*, 2000).

### Multiple sequence alignment

Gene prediction was performed using the Rice Genome Annotation Project database (RGAP, http://rice.plantbiology.msu.edu/). The major functional domains of SPL33 were predicted by the Simple Modular Architecture Research Tools (SMART) program (http://smart.embl-heidelberg.de/). Homologous sequences of *SPL33* were identified using the NCBI Blastp search program (http://www.ncbi.nlm.nih.gov/) and Phytozome (http://www.phytozome.net/). Multiple sequence alignments were conducted using the software MEGA v4.1 (http://www.megasoftware.net/) and DNAMAN v6.0 (http://www.lynnon.com/).

### Quantitative real-time PCR analysis

RNA was extracted from flag leaves, leaf sheaths, culms, and young panicles at the booting stage, and from seedling roots. For expression analysis of anti-oxidative enzyme-, photosynthesis-, and senescence-related genes, RNA was extracted from leaves of *spl33* and WT at 28 days after sowing (DAS). The total RNA extraction, reverse transcription and Real-time PCR were performed as described previously (Ma *et al.*, 2015). Primer pairs designed using GenScript (https://www.genscript.com/ssl-bin/app/primer) are listed in Supplementary Table S3. The rice *Ubiquitin* gene (*LOC_Os03g13170*) was used as a reference (primer pair *Ubi*).

### Histochemical β-glucuronidase assay

A 2724 bp promoter fragment upstream of the ATG start of *SPL33* was amplified by PCR using primers Pro-SPL33F and Pro-SPL33R (see Supplementary Table S2), and the amplicon was cloned into the *Bam*HI/*Bgl*II site of pCAMBIA1305.1. This resulting construct, p*SPL33* promoter–GUS, was introduced into WT by the *Agrobacterium*-mediated method as described previously (Jeon *et al.*, 2000). β-Glucuronidase (GUS) signals were observed as described previously (Jefferson, 1987).

### Subcellular localization

A 1965 bp *SPL33* cDNA fragment was amplified by the primers SPL33-GFP-F/R and 1305GFP-SPL33F/R (see Supplementary Table S2). The PCR product was cloned into the N-terminus of the green fluorescent protein (GFP) coding region in pAN580 and pCAMBIA1305.1-GFP vectors, to generate an *SPL33*–GFP fusion expression vector under the control of the CaMV 35S promoter using the Clonetech in-fusion PCR cloning system (TaKaRa). Transient expression constructs were co-transformed into rice protoplasts with the marker plasmid mCherry–HDEL (Nelson *et al.*, 2007), and transfected protoplasts were incubated as described previously (Chen *et al.*, 2006). GFP fluorescence was detected using a laser confocal scanning microscope (Leica TCS SP5). For transient expression in intact leaves, *A. tumefaciens* strain AH109 carrying the GFP constructs together with the p19 strain (Voinnet *et al.*, 2003) and ER marker mCherry–HDEL were infiltrated into ~5–6-week-old *Nicotiana benthamiana* leaves as described previously (Lin *et al.*, 2012).

### Pigment determination and transmission electron microscopy analysis

Equal weights of freshly collected leaves from *spl33* and WT at 28 DAS were used to determine contents of chlorophyll and carotenoids following the method of Arnon (1949). Transverse sections of leaves from plants at 28 DAS grown in paddy conditions were used for transmission electron microscopy observation as described previously (Dong *et al.*, 2013).

### Histochemical marker staining assay

Leaves of *spl33* and WT at 28 DAS were harvested for histochemical assay. Trypan blue staining for dead cells and 3,3′-diaminobenzidine (DAB) staining for H_2_O_2_ accumulation were tested as described previously (Dietrich *et al.*, 1994; Thordal-Christensen *et al.*, 1997). Callose accumulation in leaves was examined as described previously (Wolter *et al.*, 1993).

### Determination of resistance to rice blast and bacterial blight in *spl33*

Isolates of 12 *Magnaporthe oryzae* (*M. oryzae*) pathotypes virulent on WT were used to infect *spl33* plants. Inoculum preparation and seedling inoculation with the isolates followed a previously described procedure (Li *et al.*, 2008). Disease reactions were scored 7 days post-inoculation on a scale of 0–5. The disease index was calculated as described previously (Wang *et al.*, 2013).

Isolates of 11 *Xanthomonas oryzae* pv. *oryzae* (*Xoo*) pathotypes virulent on WT were used to evaluate resistance to bacterial blight in *spl33* plants. The isolates were prepared and inoculated as described by Wang *et al.* (2014). Lesion lengths were measured 2 weeks after inoculation.

### Transcriptome sequencing and data analysis

The *spl33* and WT leaves of 4-week-old plants in a growth chamber with 16 h of light at 28 °C and 8 h of darkness at 25 °C were used for total RNA extraction. RNAs were checked using an Agilent Bioanalyzer 2100 (Agilent Technologies, Waldbronn, Germany). High-quality samples with total RNA concentration ≥40 ng μl^–1^, and RNA integrity (RIN) value ≥7.0 were used for construction of cDNA libraries. The cDNA libraries were constructed using NEBNext® Ultra^TM^ RNA Library Prep Kit for Illumina® (New England Biolabs, USA) following the manufacturer’s recommendations, and index codes were added to attribute sequences to each sample. High-throughput sequencing was performed on an Illumina HiSeq 2500 (version 4.0) genome analyser (Illumina, San Diego, CA, USA). The 150 bp paired-end reads generated from *spl33* and WT plants were processed as described previously (Hiremath *et al.*, 2011). In total 27 892 108 and 27 909 005 clean reads were obtained for *spl33* and WT, respectively. Clean reads were aligned to the Rice Genome Annotation Project (http://rice.plantbiology.msu.edu/) using Bowtie2 (Langdon, 2015). Differentially expressed genes (DEGs) and transcript expression analyses were performed using Cufflinks (Trapnell *et al.*, 2012), DEseq2 (Love *et al.*, 2014) and EdgeR (Robinson *et al.*, 2010). DEGs were determined by at least two packages, with an absolute log2-fold change value ≥1 and a false discovery rate ≤0.001. Gene Ontology (GO) annotations of DEGs were obtained from the Rice Genome Annotation Project. GO functional enrichment analysis and Kyoto Encyclopedia of Genes and Genomes (KEGG) analysis were performed as described previously (Tang *et al.* 2013).

## Results

### Characterization of the *spl33* mutant

**Fig. 1.**
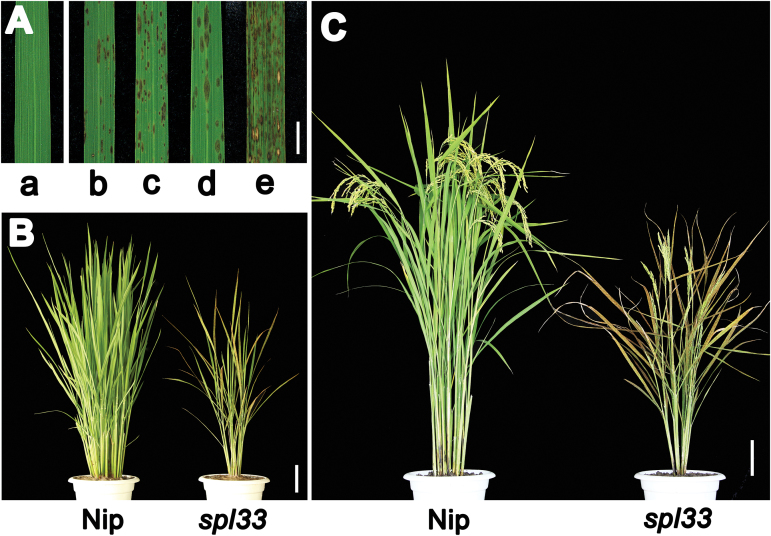
Comparison of wild type (Nip) and *spl33* plants. (A) Lesion mimic phenotype in *spl33*. a, clean leaf blade in the Nipponbare wild type (WT); b and c, lesion mimics at seedling and tillering stages of greenhouse-grown *spl33* in winter; d and e, lesion mimics at seedling and tillering stages of field-grown *spl33* in summer. (B) *spl33* plant at tillering stage showing early senescence in *spl33*. (C) WT and *spl33* plants at heading stage. Note that *spl33* was full of lesion mimics. Nip, Nipponbare. Scale bar: 1cm in (A), 10 cm in (B, C).

Small, reddish-brown lesions started to appear on the leaves of three-leaf-stage *spl33* mutant seedlings (about 20 DAS) and continued to form throughout ripening growth stages (Fig. 1A–C). Notably, newly emerging leaves showed no lesions and were indistinguishable from wild-type (WT) Nipponbare (Nip), but gradually developed lesions as they expanded (Fig. 1A). The *spl33* phenotype was seen under both field conditions in summer (25–35 °C) and greenhouse conditions in winter (25–30 °C) (Fig. 1A). Young seedlings displayed fewer and relatively small reddish-brown lesions on fully expanded leaves; the lesions increased in both number and size at the early flowering stage, followed by leaf chlorosis and rapid senescence of the whole plant at the late flowering stage, while the leaves of WT plants stayed green (Fig. 1B, C). In addition, tiller number, plant height, panicle length, total grain number per plant, spikelet fertility and 1000-grain weight of the *spl33* mutant were all significantly decreased relative to WT plants (see Supplementary Fig. S1).

To determine whether the lesion phenotype of the *spl33* mutant was affected by environmental factors, such as light, we performed light avoidance assays on the *spl33* mutant and WT. In the light avoidance experiment, part of the newly emerging leaves was covered with aluminum foil to block light while the uncovered leaf areas remained exposed to light. The leaf area without exposure to light did not develop lesions of any size in contrast to the neighboring uncovered areas, which developed scattered lesions (see Supplementary Fig. S2), indicating that lesion formation in *spl33* is light-dependent and that light-induced lesion-related signals are not transported across the leaf. Thus, we identified an HR-mimicking mutant, *spl33*, that develops lesions in a light-dependent manner.

### Map-based cloning of the *SPL33* locus

Reciprocal crosses were made between the *spl33* mutant and WT. The F_1_ plants exhibited WT phenotype and the segregation of F_2_ populations fitted a phenotype ratio of 3 WT:1 mutant indicating that the *spl33* phenotype was caused by a single recessive nuclear gene mutation (see Supplementary Table S4).

**Fig. 2.**
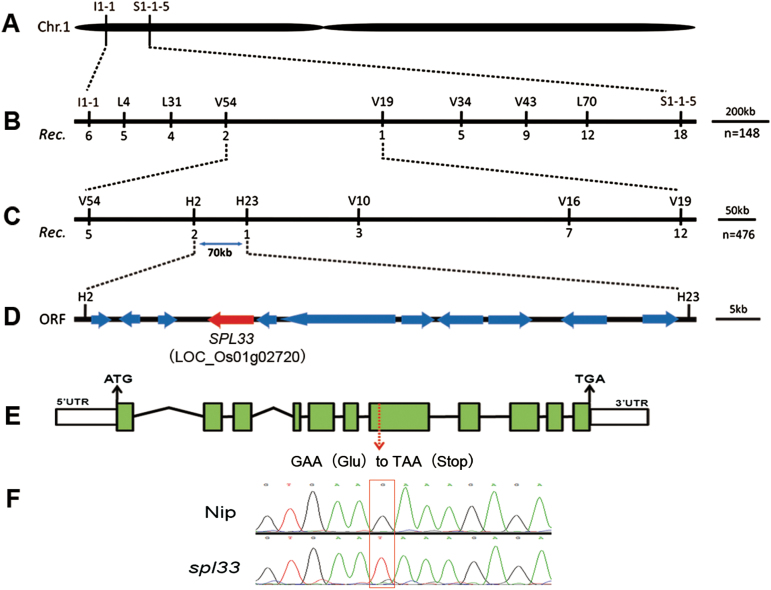
Genetic and physical maps of the *SPL33* gene. (A) The *SPL33* gene was located on chromosome 1 between InDel markers I1-1 and S1-1-5. (B) The *SPL33* gene was delimited to the V54-V19 interval using 148 F_2_ mutant individuals; marker names and number of recombinants are shown. (C) Fine genetic mapping of the *SPL33* gene based on 476 mutant F_2_ individuals. (D) Eleven putative ORFs were located in an ~70-kb region. (E) Gene structure *LOC_Os01g02720*. Eleven exons and ten introns are indicated by green rectangles and black lines, respectively; a G to T point mutation was identified in the seventh exon (red arrow) generating a premature termination codon. (F) Sequence analysis of the G-to-T mutation site in plants of wild type and *spl33*.

For mapping of the *spl33* locus, an F_2_ population from the cross of *spl33* × *indica* cv. Dular was used. When 20 F_2_ individuals with mutant phenotype and 20 with WT phenotype were genotyped using polymorphic markers selected from 212 insertion and deletion (InDel) markers (Lei *et al.*, 2013), we localized the mutation locus between the InDel markers I1-1 and S1-1-5 on chromosome 1 (Fig. 2A). With further genotyping on 148 mutant F_2_ individuals derived from the same cross with seven additional polymorphic markers, the *spl33* locus was delimited to the V54–V19 interval (Fig. 2B). Using an additional 476 F_2_ mutant individuals and four newly developed polymorphic markers, we finally anchored *spl33* to a 70 kb region between InDel markers H2 and H23 (Fig. 2C). Eleven open reading frames (ORFs) were predicted in this region (Fig. 2D and Supplementary Table S5) (http://rice.plantbiology.msu.edu/). Sequencing and comparison of those ORFs cloned from *spl33* and WT revealed that the fourth ORF (*LOC_Os01g02720*) had a single-base substitution (G^2493^→T^2493^) in its seventh exon, leading to a premature stop (Fig. 2E, F), thus placing *LOC_Os01g02720* as a candidate position for *SPL33*.

### Functional complementation of the *spl33* mutant with *LOC_Os01g02720*

**Fig. 3.**
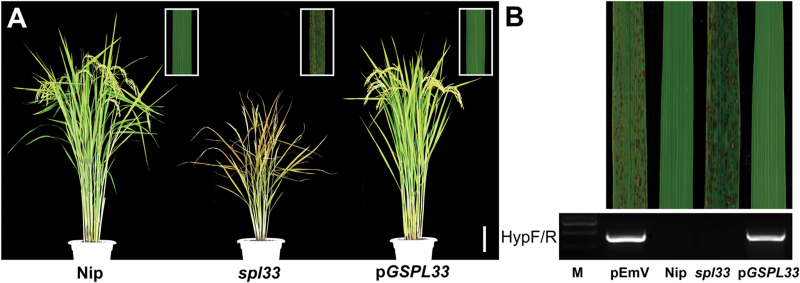
Genetic complementation of *spl33*. (A) *spl33* plant transformed with the genomic sequence of *SPL33* (p*GSPL33*) was completely recovered to the wild type Nipponbare (Nip) phenotype. The insert indicates enlargement of leaf section with lesion spots. (B) Transgenic plants were verified by the presence of the hygromycin selectable marker gene. M, molecular markers; pEmV, the empty vector. Scale bar: 10 cm in (A).

To verify whether the single base substitution in *LOC_Os01g02720* was responsible for the *spl33* phenotype, we constructed the vector pG*SPL33* that contains a WT-derived 9881 bp genomic DNA fragment consisting of the entire *SPL33* coding region, 2732 bp upstream and 2851 bp downstream sequences, and introduced it into *spl33* by *A. tumefaciens*-mediated transformation. The corresponding empty vector pEmV was also transformed as a control. Of 72 regenerated T_0_ plants, 64 were positive transformants, and all of them exhibited a complete rescue of the mutant phenotype (Fig. 3A), whereas none of the plants transformed with the control vector displayed the WT phenotype (Fig. 3B). The rescued phenotype was seen again in T_1_ plants positive for the transgene (see Supplementary Fig. S3). Meanwhile, we also introduced an overexpression vector (p*Ubi::SPL33*) containing the entire cDNA sequence of *SPL33* into the *spl33* mutant. Normal green phenotypes were also completely recovered in 32 independent p*Ubi::SPL33* transgenic lines (see Supplementary Fig. S4). Taken together, those results demonstrate that *LOC_Os01g02720* is *SPL33* and that the single-base substitution G^2493^→T^2493^ in *spl33* causes the lesion-mimic phenotype.

### *SPL33* encodes an eEF1A-like protein

**Fig. 4.**
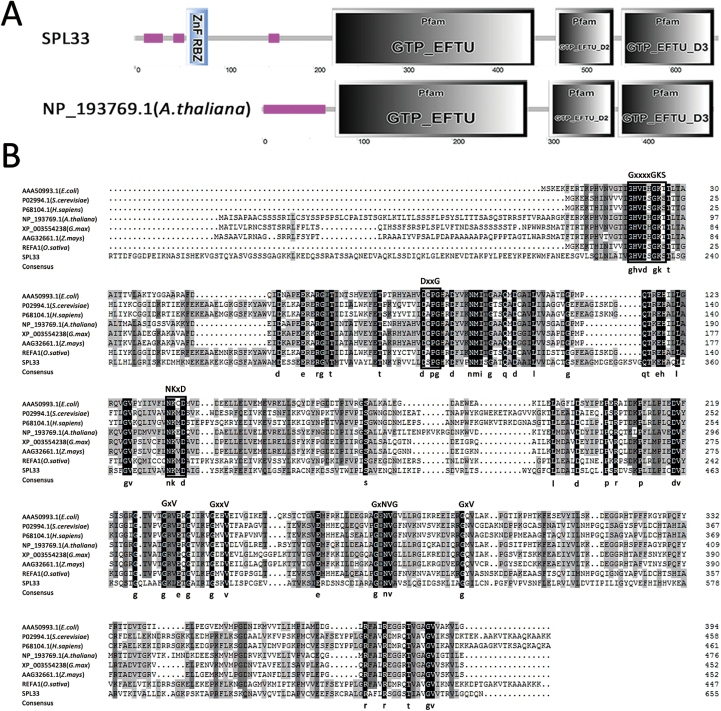
*SPL33* encodes a eukaryotic translation elongation factor1A (eEF1A)-like protein. (A) Predicted domains of *SPL33* by Simple Modular Architecture Research Tools (SMART). *SPL33* has a zinc finger domain (blue rectangle) at the N-terminus. (B) Alignment of the conserved motifs of eukaryotic translation elongation factor 1 alpha (eEF1A) and prokaryotic elongation factor (EF-Tu) from multiple organisms. The amino acid sequence alignment indicated that eEF1A is a highly conserved domain. The region corresponding to the GTP/GDP binding domain of GTP-binding proteins is indicated by white boxes, the conserved amino acid sequences are shown at the top, and the consensus amino acid residues are shown at the bottom.

Sequence comparison between genomic DNA and cDNA showed that *SPL33* is composed of 11 exons separated by 10 introns (Fig. 2E). The coding sequence (CDS) of *SPL33* consists of 1968 nucleotides, and encodes a putative 655 amino acid protein with a molecular mass of 71 kDa. *SPL33* was predicted to encode an eEF1A-like protein, containing a zinc finger domain, a GTP-binding domain (GTP_EFTU), and two oligonucleotide binding domains referred to as domain 2 (GTP_EFTU_D2) and domain 3 (GTP_EFTU_D3) (Fig. 4A). GTP_EFTU_D2 adopts a β-barrel structure, and is involved in binding to an aminoacyl-tRNA. GTP_EFTU_D3 is involved in binding to both aminoacyl-tRNA and eukaryotic factor 1 beta (eEF1B). The N-terminal of *SPL33* also has a zinc finger domain that is not present in other eEF1As, such as *Arabidopsis* eEF1A (Fig. 4A). Comparison of the amino acid sequences of *SPL33* with those orthologs from other organisms showed that the three regions significant for binding of GTP and hydrolysis of GTP to GDP (Bourne *et al.*, 1991), and some other active amino acid sites, are highly conserved in all aligned eEF1A or EF-Tu (Fig. 4B).

**Fig. 5.**
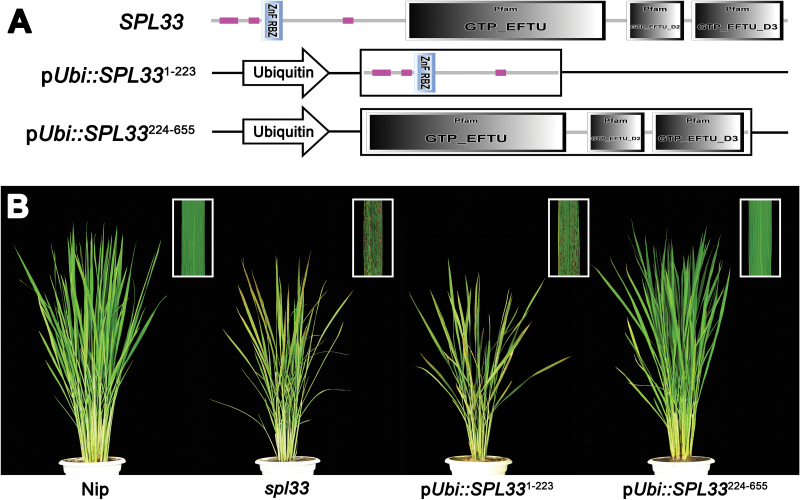
Dissection of *SPL33* by transformation. (A) Schematic diagram of the overexpression vectors. p*Ubi::SPL33*^1–223^: the 1–669 bp sequence of *SPL33* (*SPL33*^1–223^, encoding amino acids 1–223 of SPL33) that contains the zinc finger domain under the control of the ubiquitin promoter; p*Ubi::SPL33*^224–655^: the 670–1968 bp sequence of *SPL33* (*SPL33*^224–655^, encoding amino acids 224–655 of SPL33) that contains three EF-Tu structural domains under the control of the ubiquitin promoter. (B) Phenotypes of wild-type (Nip), *spl33* mutant, and two transgenic T_0_ plants carrying p*Ubi::SPL33*^1–223^ and T_0_ p*Ubi::SPL33*^224–655^, respectively. The inset indicates enlargement of leaf section with lesion spots.

To further confirm the functions of different domains of *SPL33*, we performed complementation tests by separately transforming the 1–669 bp sequence of *SPL33* (*SPL33*^1–223^, encoding the first 223 amino acids) containing the zinc finger domain and the 670–1968 bp sequence of *SPL33* (*SPL33*^224–655^, encoding the remaining 432 amino acids) containing the three EF-Tu domains, both under control of the *ubiquitin* promoter, into the *spl33* mutant. The mutant phenotype was completely rescued in the p*Ubi::SPL33*^224–655^ positive plants, whereas the p*Ubi::SPL33*^1–223^ vector failed to complement the *spl33* mutant phenotype (Fig. 5). These results indicated that the C-terminal region of SPL33 containing the three EF-Tu domains is enough for functioning, whereas the N-terminal zinc finger domain is redundant, at least for preventing rice leaves from showing lesion mimics.

### Expression pattern analysis of *SPL33*

**Fig. 6.**
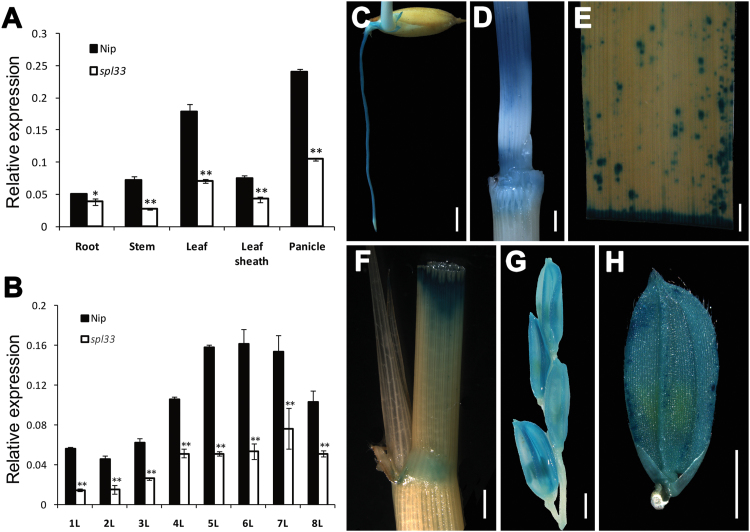
Expression analysis of *SPL33*. (A) Expression of *SPL33* in roots of 7-day-old seedling, stem, flag leaf blade and sheath, and young panicle at booting stage of wild type Nipponbare (Nip) and mutant *spl33* analysed by quantitative RT-PCR. (B) Developmental expression pattern of *SPL33* in different leaves along plant growth. 1L–8L represent the first to eighth leaf, respectively. (C–H) Histochemical signals in plants carrying the *SPL33* promoter–GUS reporter gene. GUS signals were detected in the root (C), stem (D), leaf (E), leaf sheath (F), panicle (G), and spikelet (H). Scale bar: 2 mm in (C–H). Error bars in (A, B) indicate standard deviations of three independent samples. Data are means±SD of three biological replicates (Student’s *t*-test: *P<0.05; **P<0.01).

Quantitative real-time PCR (qRT-PCR) analysis of *SPL33* in various organs showed that *SPL33* was ubiquitously expressed in all organs examined (Fig. 6A). The strongest expression was detected in panicles and leaves, with relatively weak expression in roots, stems and leaf sheaths. Expression levels in all these organs in the *spl33* mutant were significantly decreased relative to those of the wild-type (Fig. 6A). In addition, the expression level of *SPL33* differed in leaves at different growth stages, and peaked in the sixth leaf (Fig. 6B). We generated transgenic plants of p*SPL33::GUS* expressing the *GUS* reporter gene driven by the native promoter of *SPL33* to more precisely examine the spatial and temporal expression patterns of *SPL33*. Consistent with the qRT-PCR results, we observed GUS signals in all tissues of transgenic plants (Fig. 6C–H). These results indicated that *SPL33* is broadly expressed in all organs and at all developmental stages examined.

### Subcellular localization of SPL33 protein

**Fig. 7.**
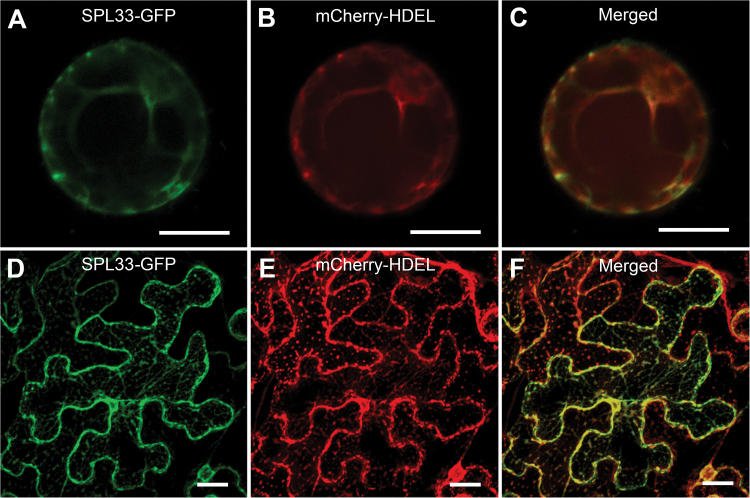
Subcellular localization of SPL33 protein. (A–C) Rice protoplast transient assay. (D–F) *N. benthamiana* leaf assay. *SPL33*–GFP, SPL33 fused to GFP; mCherry–HDEL, endoplasmic reticulum (ER) marker. Scale bar: 20 μm.

To determine the subcellular localization of *SPL33*, the full-length coding sequence of *SPL33* was fused to the N-terminus of green fluorescent protein (GFP). When transiently expressed in rice protoplasts, the GFP signal was co-localized with the ER marker mCherry–HDEL (Nelson *et al.*, 2007) (Fig. 7A–C). To validate this observation, we transformed the plasmid containing *SPL33*–GFP fusion into *N. benthamiana* leaves. SPL33–GFP protein was exclusively detected in the ER (Fig. 7D, E). These results indicated that the SPL33 protein is localized to the ER.

### The *SPL33* regulates ROS accumulation and cell death in rice

**Fig. 8.**
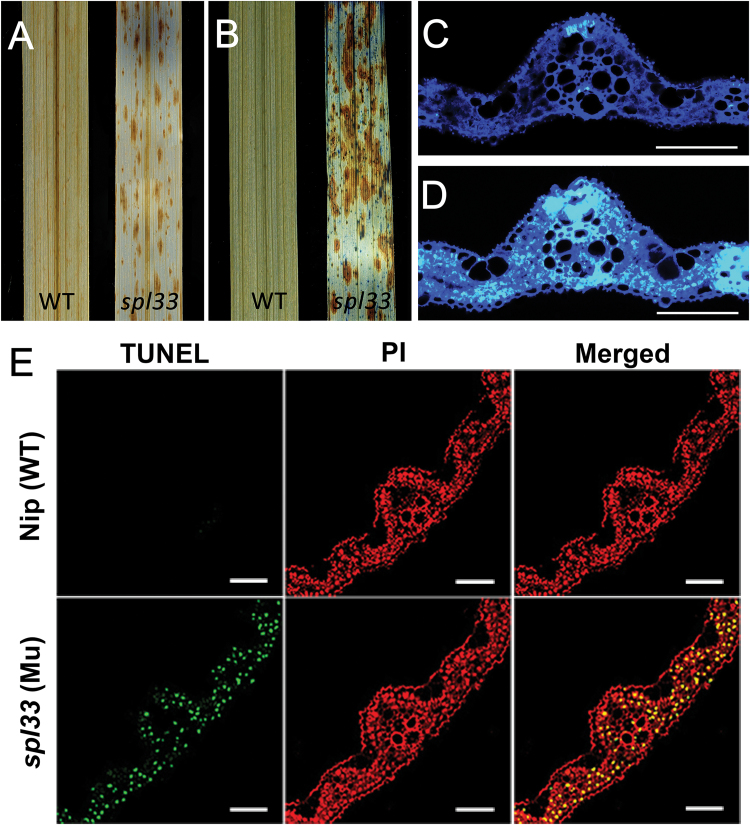
H_2_O_2_ accumulation and PCD detection in *spl33*. (A) DAB staining for H_2_O_2_ accumulation. (B) Trypan blue staining for cell death. (C, D) Aniline blue staining for callose accumulation under UV light; fluorescent regions indicate callose accumulation. (C) WT; (D) *spl33.* Scale bar: 100 μm. (E) DNA fragmentation detection in mesophyll cells by TUNEL assay. Red signal represents staining with propidium iodide, and yellow and green signals indicate TUNEL-positive nuclei of dead cells resulting from PCD. Scale bar: 100 μm. WT and *spl33* leaf samples in (A–E) were analysed at 28 DAS.

Reactive oxygen species (ROS) are major signaling molecules in plant PCD, and high concentrations of ROS, including superoxide and hyperoxide, may cause cellular damage or trigger PCD (Khanna-Chopra, 2012). DAB is an indicator of H_2_O_2_ accumulation (Wang *et al.*, 2015*a*). Using DAB staining, we found that the *spl33* mutant had much stronger H_2_O_2_ accumulation than WT (Fig. 8A). Typan blue staining, a traditional method for selective staining of dead tissues or cells (Qiao *et al.*, 2010), consistently showed that the *spl33* mutant had a large number of blue spots after staining, whereas the wild-type did not (Fig. 8B). In addition, callose, a plant polysaccharide component of primary cell walls induced by wounding or pathogen infection (Qiao *et al.*, 2010), accumulated at a much higher level in vascular bundles, sclerenchyma and mesophyll cells of *spl33* leaves than in WT (Fig. 8C, D). Together, our results suggested that the reddish-brown lesions in the *spl33* mutant were caused by ROS accumulation and irreversible membrane damage. Further, we performed a terminal deoxynucleotidyl transferase dUTP nick end labelling (TUNEL) assay for detection of DNA fragmentation, a hallmark of programmed cell death (Kim *et al.*, 2011). TUNEL signals in nuclei were strong and randomly distributed in the *spl33* mutant, whereas no TUNEL signals were detected in WT (Fig. 8E). These results suggested that loss of function of *SPL33* triggers the PCD pathway, ultimately leading to development of the *spl33* lesion-mimic phenotype.

To further understand the biochemical mechanism involved in ROS accumulation in the *spl33* mutant we undertook expression analyses of the genes encoding anti-oxidative enzymes superoxide dismutase (SOD), catalase (CAT) and peroxidase (POD) in *spl33* mutant and WT. These three enzymes are normally activated to remove ROS under oxidative stress (Miller *et al.*, 2010). However, the expressions of both *CAT* and *POD* in the *spl33* mutant were not obviously changed, even though the expression of *SOD* in the *spl33* was elevated by almost two-fold (see Supplementary Fig. S5). Those results suggested that loss-of-function of *SPL33* activated *SOD*, which might remove some superoxide, but not CAT and POD, which might result in insufficient capability for scavenging excessive H_2_O_2_.

### *SPL33* regulates early leaf senescence in rice

Early leaf senescence is a consequence of uncontrolled PCD, and has been described as a characteristic of some LMMs (Wang *et al.*, 2015*b*). To examine early leaf senescence in the *spl33* mutant, we measured four indicators of senescence, namely chloroplast structure (Wang *et al.*, 2015*b*), chlorophyll content (Jiao *et al.*, 2012), photosynthesis-related gene expression (Lim *et al.*, 2007), and senescence-related gene expression (Park *et al.*, 2007).

**Fig. 9.**
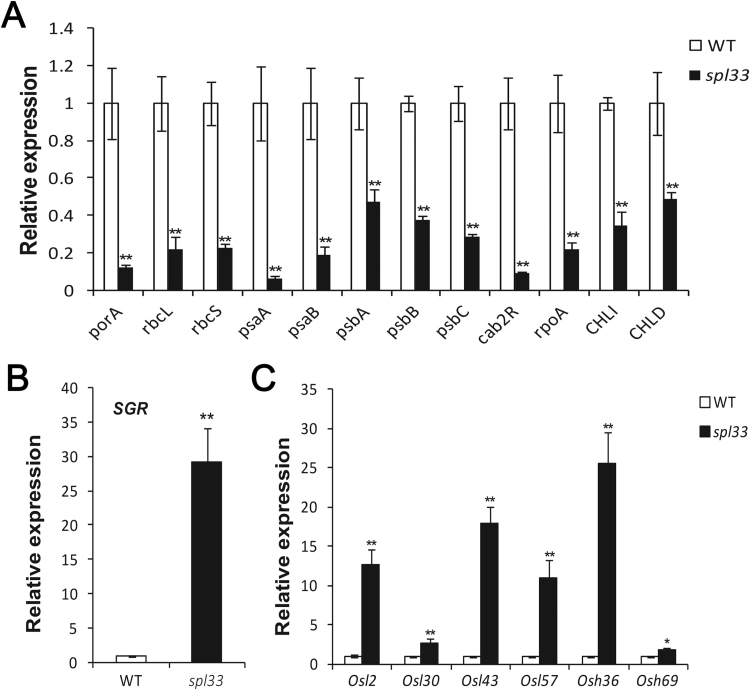
Identification of early leaf senescence in *spl33* at the molecular level. Relative expression of photosynthesis-related genes (A), *STAY GREEN* (*SGR*) gene (B), and senescence-associated genes (C). Wild-type (WT) and *spl33* leaves were collected from 28-day-old seedling; the expression level of each gene in WT was normalized to 1. Data are means±SD of three biological replicates (Student’s *t*-test: *P<0.05; **P<0.01).

Examination of ultra-structures of *spl33* and WT mesophyll cells in leaves of 28-day-old seedlings by transmission electron microscopy showed that chloroplasts in mesophyll cells of WT leaves were fully developed with intact membranes, whereas mesophyll cells surrounding the spotted areas of *spl33* leaves were completely disrupted, leaving membrane-bound bodies in the cytoplasm (see Supplementary Fig. S6A–D. Coincidently, chlorophyll (a, b) and carotenoid contents were significantly decreased in *spl33* leaves (Supplementary Fig. S6E). These results implied that irreversible degradation of chloroplasts and decreased photosynthesis in *spl33* leaves could be responsible for the early leaf senescence. On the other hand, qRT-PCR of 12 photosynthesis-related genes revealed that the expression levels of *porA*, *rbcL*, *rbcS*, *psaA*, *psaB*, *psbA*, *psbB*, *psbC*, *cab2R*, *rpoA*, *CHLI*, and *CHLD* in *spl33* were significantly down-regulated to 0.12-, 0.22-, 0.23-, 0.07-, 0.19-, 0.47-, 0.38-, 0.29-, 0.09-, 0.22-, 0.35-, and 0.49-fold, respectively, of those in WT (Fig. 9A). qRT-PCR of the senescence-induced *STAYGREEN* (*SGR*) gene and six senescence-associated genes revealed that the expression levels of *SGR*, *Osl2*, *Osl30*, *Osl43*, *Osl57*, *Osh36*, and *Osh69* were dramatically up-regulated, being 29.2-,12.6-, 2.8-, 17.9-, 10.9-, 25.6-, and 1.8-fold higher than in comparative WT leaves (Fig. 9B, C). These results provided molecular evidence for the early leaf senescence in *spl33* plants. We concluded that *SPL33* may play an important role in regulating leaf senescence, and that its loss-of-function may accelerate early leaf senescence.

### *SPL33* regulates defense responses in rice

**Fig. 10.**
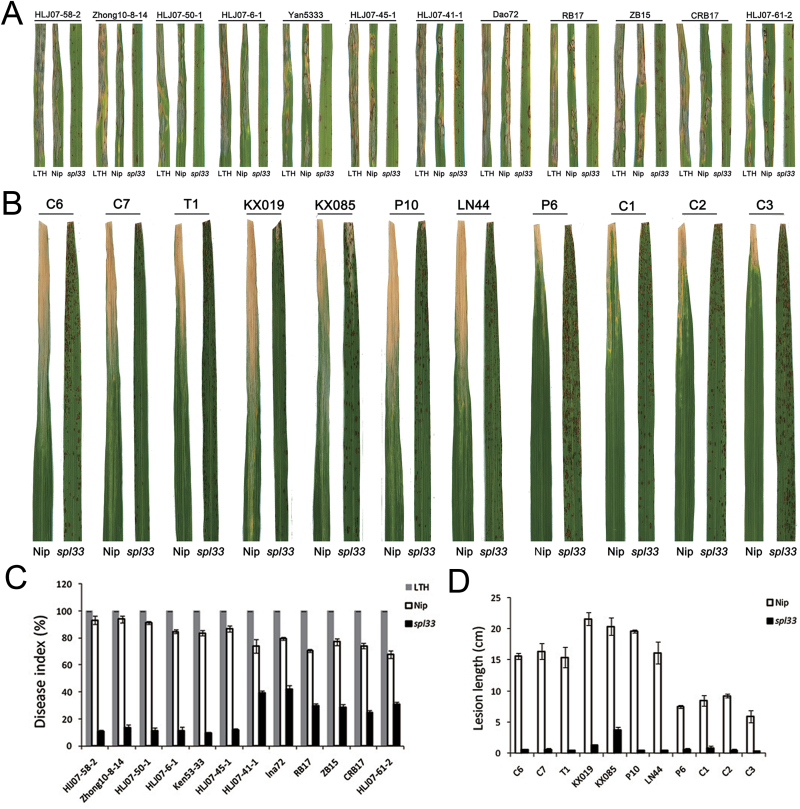
Enhanced resistance in the *spl33* mutant to *Magnaporthe oryzae* and *Xanthomonas oryzae* pv. *oryzae* isolates. (A) Reactions to 12 *M. oryzae* isolates. The variety LTH was used as a susceptible control. (B) Reactions to 11 *Xoo* isolates. (C) Disease indices of WT and *spl33* to *M. oryzae* isolates. (D) Lesion lengths of wild-type Nipponbare (Nip) and *spl33* to 11 *Xoo* isolates. Data are means±SD of 15 plants.

The lesion-mimic phenotype in *spl33* plants resembles the HR that occurs in plants following infection by many pathogens. Some LMMs show enhanced resistance to fungal and bacterial pathogens (Fekih *et al.*, 2015; Wang *et al.*, 2015*b*). To examine whether the *spl33* mutant also gains disease resistance, we inoculated the mutant and the wild-type plants with isolates of 12 *M. oryzae* pathotypes and 11 *Xoo* pathotypes virulent against the WT. The *spl33* plants exhibited significantly enhanced resistance to all tested isolates of both pathogens (Fig. 10).

Defense-response genes were activated during lesion development in some rice LMMs (Fekih *et al.*, 2015; Wang *et al.*, 2015*b*). To determine whether and when defense-response genes were activated in the *spl33* mutant, we examined the expression of defense-response marker genes *PR1a*, *PBZ1* and *PO-C1* (Shirsekar *et al.*, 2014; Wang *et al.*, 2015*b*) in both the mutant and WT plants by qRT-PCR at 14 DAS (before appearance of visible lesions in *spl33* plants), 21 DAS (lesions just visible) and 28 DAS (lesions clearly visible). At 14 DAS expression levels of *PR1a*, *PBZ1* and *PO-C1* in the *spl33* mutant were 2.14-, 1.11-, and 6.66-fold, respectively, of those in WT plants; at 21 DAS expression of the three genes in *spl33* was 199.17-, 86.47-, and 296.52-fold higher, respectively, than WT; and at 28 DAS their expression dropped but was still higher than WT by 5.44-, 8.57-, and 19.83-fold, respectively (see Supplementary Fig. S7). Together, these results suggest that lack of the eEF1A-like protein triggers the defense response, which leads to enhanced disease resistance associated with lesion formation in *spl33*.

### Transcriptome sequencing suggests that *SPL33* may have multiple functions

*SPL33* encodes an eEF1A-like protein, known to be involved in PCD, nuclear export, proteasome-mediated degradation of damaged protein, and actin-binding and bundling besides a central role in translation (Migliaccio *et al.*, 2015). However, almost all those known functions were documented from studying Saccharomycetes and mammals, with scant knowledge of their role in plants (Gao *et al.*, 2013). In order to investigate the functions of the eEF1A-like protein encoded by *SPL33* in rice, we performed transcriptome sequencing of both the *spl33* mutant and WT at 28 DAS when mutant plants were exhibiting multiple lesions.

Based on the RNA-seq data, there were 4792 DEGs between the *spl33* mutant and WT, of which 3867 were up-regulated and 925 down-regulated (see Supplementary Dataset S1). All the reliable DEGs were then analysed for GO functional enrichment. Genes involving translation (GO:0006412), structural molecule activity (GO:0005198) and ribonucleoprotein complex (GO:0030529) were significantly enriched in *spl33*, suggesting that *SPL33* may be involved in protein biosynthesis. Genes involved in response to biotic stimulus (GO:0009607), response to stress (GO:0006950), and cell death (GO:0008219) were significantly enriched among the up-regulated group (Supplementary Dataset S2, Up-regulated). GO terms associated with photosynthesis (GO:0015979) and signal transduction (GO:0007165) were among the down-regulated genes (Supplementary Dataset S2, Down-regulated). These results of GO enrichment were consistent with the earlier results, suggesting that *SPL33* functions in disease response and photosynthesis in addition to protein translation.

To further explore the biological pathways in which *SPL33* may be involved, we performed KEGG enrichment analysis for the DEGs between the *spl33* mutant and WT. Seven pathways were significantly (P<0.01) enriched for up-regulated genes and four for down-regulated ones (see Supplementary Dataset S3). The highly enriched up-regulated pathways were mainly related to protein synthesis (ribosome, biosynthesis of amino acids and ribosome biogenesis in eukaryotes), PCD and defense response (Supplementary Dataset S3, Up-regulated). The highly enriched pathways with down-regulated genes were related to photosynthesis (photosynthesis–antenna proteins, photosynthesis–porphyrin and chlorophyll metabolism) (Supplementary Dataset S3, Down-regulated). These results suggest that *SPL33* is involved in protein translation, and regulation of cell death, leaf senescence, and defense response, consistent with the results of GO enrichment.

The enriched plant–pathogen interaction pathway involved 45 genes, among which 43 were significantly up-regulated and two were significantly down-regulated in *spl33* (see Supplementary Dataset S4). Up-regulation involved genes that participate in multiple signaling-related events in innate immunity (Supplementary Fig. S8 and Supplementary Dataset S4). Examples include the pattern recognition receptor (PPR) *OsCEBiP* involved in recognition of pathogen- or microbe-associated molecular patterns; *MEKK1* and two *MKK*s involved in phosphorylation of kinase cascades; *CNGC*, three *CPK*s, three *CAM*s and seven *CML*s involved in calcium signaling; *Rboh* involved in generation of ROS as an NADPH oxidase; rice homologs of *RPM1* and *RPS2* involved in recognition of avirulence effectors produced by pathogens; and two *PR1*s that serve as antimicrobial proteins. The up-regulated receptor genes and signaling molecules in the *spl33* mutant may induce innate immunity, cell wall reinforcement, antimicrobial proteins and phytoalexin accumulation, all of which have been implicated in disease response.

Further analysis on the 4792 DEGs identified eight salicylic acid (SA) biosynthesis-related *PAL* genes, 21 jasmonic acid (JA) biosynthesis-associated genes, and 3 ethylene (ETH) biosynthesis-associated genes. Interestingly, all but 2 of them were up-regulated in *spl33* (see Supplementary Table S6). Those results indicate that the eEF1A-like gene plays a negative role in both SA and JA/ETH biosynthesis and that its loss-of-function may result in higher SA and JA/ETH accumulation, thus enhancing the plant defense network.

## Discussion

### *SPL33* encodes an eEF1A-like protein containing a zinc finger domain in addition to three conserved EF-Tu domains

LMMs are important for understanding PCD and defense responses in plants. In the present study, we isolated an LMM, *spl33*, from a tissue culture-derived population of cv. Nipponbare. The mutant displayed small reddish brown lesions on the leaves from the seedling stage, early leaf senescence at the late flowering stage, semi-dwarfness, fewer tillers and reduced spikelet fertility (Fig. 1). The *SPL33* gene was identified as *LOC_Os01g02720*, encoding an eEF1A-like protein. Functional complementation with wild-type *LOC_Os01g02720* rescued all mutant phenotypes observed in *spl33* (Fig. 3 and Supplementary Fig. S4). It is worth noting that the SPL33 protein contains a zinc finger domain in addition to three highly conserved EF-Tu domains of typical eEF1As present in a wide range of organisms (Fig. 4). Complementation tests by expressing truncated SPL33 in the *spl33* mutant confirmed that only the three EF-Tu domains were necessary for *SPL33* function, whereas the zinc finger domain was dispensable (Fig. 5). This suggests that *SPL33* could fulfill the functions of typical eEF1As. Transcriptome sequencing of *spl33* and WT showed that both protein translation-related GO terms and protein synthesis-related KEGG pathways were highly enriched (Supplementary Datasets S2 and S3), indicating that *SPL33* was involved in protein translation, the canonical role of typical eEF1As.

There are at least ten other distinct rice genes that are predicted to encode putative eEF1As (see Supplementary Fig. S9). The SPL33 protein shares 97.26% amino acid identity with LOC_Os04g50870, which is located on chromosome 4, which was also predicted to contain a zinc finger domain, and has 17.58–49.24% amino acid identity with the other nine putative eEF1A proteins (Supplementary Fig. S9). There was no sequence variation of *LOC_Os04g50870* between WT Nipponbare and the *spl33* mutant (data not shown). Expression analysis showed that *LOC_Os04g50870* was expressed at relatively low levels in both *spl33* mutant (about 1/7 of *SPL33*) and WT plants (about 1/31 of *SPL33*) compared with *SPL33* (Supplementary Fig. S10). In addition, the expression level of *LOC_Os04g50870* in WT plants was only half of that in the mutant plants, which is contrary to that the expression level of *SPL33* in the wild-type plants was 2.1-fold of that in the mutant plants (Supplementary Fig. S10). These results suggest that the two homolog genes, *SPL33* and *LOC_Os04g50870*, seem to have no functional redundancy in regulating the mutant phenotypes. Further investigation is needed to determine the relationship between *SPL33* and *LOC_Os04g50870*.

The *spl33* allele in the mutant has a single-base G^2493^→T^2493^ substitution that causes a premature stop of the SPL33 protein (Fig. 2), and it is loss-of-function of *SPL33* that induces the mutant phenotypes including spotted leaves, early leaf senescence and enhanced resistance to *M. oryzae* and *Xoo*. To our knowledge, this is the first report of an eEF1A-like mutant in plants, and also the first report that an eEF1A-like protein is involved in cell death and the defense response in plants.

### Cell death and early leaf senescence are caused by loss-of-function of *SPL33*

The *spl33* mutant exhibited spontaneous leaf spotting starting from the young seedling stage, mimicking the localized HR that occurs following infection by many pathogens. Our study revealed that mutation of the eEF1A-like protein encoded by *SPL33* was responsible for the mutant phenotype. Even though eEF1As have been reported to function in PCD in higher vertebrates (Migliaccio *et al.*, 2015), there were no reports describing a similar function in plants. In order to determine whether the eEF1A-like protein encoded by *SPL33* controls PCD in plants, we performed expression analyses of several histochemical markers using staining methods, including DAB staining for H_2_O_2_ accumulation, trypan blue staining for cell death, aniline blue staining for callose accumulation and propidium iodide staining for TUNEL-positive nuclei of PCD cells (Qiao *et al.*, 2010; Kim *et al.*, 2011; Fekih *et al.*, 2015; Wang *et al.*, 2015*a*), and the transcriptome sequencing in *spl33* and wild-type plants, respectively. All the results obtained (Fig. 8 and Supplementary Datasets S2 and S3) provided evidence that the eEF1A-like protein controls PCD in plant. Considering that the *spl33* mutation is characterized by spontaneous cell death in leaves, we thus concluded that *SPL33* serves as a negative regulator of PCD-mediated cell death in rice.

Early leaf senescence also occurred in the *spl33* mutant, and this phenotype was obviously accelerated at the tillering and flowering stages (Fig. 1). To date, only a few studies have described senescence as a phenotype of LMM plants, and the senescence syndromes were not well analysed in those mutants (Qiao *et al.*, 2010; Wang *et al.*, 2015*b*). Therefore, it was necessary to determine that early leaf senescence does occur in the *spl33* mutant. For this purpose, we selected four indicators for leaf senescence, namely loss of chlorophyll (Jiao *et al.*, 2012), breakdown of chloroplasts (Wang *et al.*, 2015*b*), down-regulation of photosynthesis-related genes (Wang *et al.*, 2015*b*), and up-regulation of senescence-associated genes (Park *et al.*, 2007), and performed relevant senescence assays. All results (Supplementary Fig. S6 and Fig. 9) suggest that early leaf senescence happens in *spl33* plants, and this was further verified by the transcriptome sequencing of the *spl33* mutant and WT (Supplementary Datasets S2 and S3). Since loss-of-function of *SPL33* resulted in early leaf senescence we deduced that the eEF1A-like protein encoded by *SPL33* may function in regulation of leaf senescence.

The PCD-mediated leaf senescence is generally regarded to have a more complex physiological basis than cell death in that the senescence is normally characterized by nutrient remobilization between organs and is an integral part of normal plant development (Rogers, 2015). It is worth noting that the PCD-mediated cell death and early leaf senescence seem to occur simultaneously in *spl33* plants (Fig. 1A–C). In order to better dissect the regulatory mechanisms for cell death and leaf senescence, it is necessary to investigate whether cell death and early leaf senescence are a continuum or distinct processes, or regulated separately in *spl33* plants.

### Defense responses are induced by loss-of-function of *SPL33*

Defense response genes may be activated during the development of symptoms in lesion-mimic mutants, and may contribute to enhanced resistance to pathogens (Wang *et al.*, 2015*b*). The loss-of-function of *SPL33* results in induced HR-like lesions. Enhanced resistances to blast and bacterial blight were observed in the *spl33* mutant (Fig. 10). Defense responses can also involve activation of PR genes and *PR1a*, *PBZ1* and *PO-C1* have been used as molecular markers for monitoring defense responses in rice. These three PR genes were all significantly up-regulated in *spl33* plants during the development of lesion mimics (see Supplementary Fig. S7), and may have been involved in enhancing the resistances. The induced resistance in *spl33* appears to be broad-spectrum and non-specific (Fig. 10).

KEGG enrichment analysis suggested that the plant–pathogen interaction pathway was constitutively activated in the *spl33* mutant (Supplementary Fig. S8 and Supplementary Dataset S4). A number of genes involved in pathogen-associated molecular pattern (PAMP)-triggered immunity (PTI) were significantly up-regulated, including pattern recognition receptors, e.g. *OsCEBiP* (Akamatsu *et al.*, 2013); MAP kinase kinases, e.g. *OsMPK3*, *OsMKK4*, and *OsMPK6* (Kishi-Kaboshi *et al.*, 2010; Hu *et al.*, 2015); MEK kinase 1, e.g. *OsMKK1* (Kumar *et al.*, 2008); cyclicnucleotide-gated channels, e.g. *CNGC10* (Guo *et al.*, 2008); calmodulin-like proteins, e.g. *OsMSR2* (Xu *et al.*, 2013); calcium-dependent protein kinases, e.g. *OsCPK10* (Fu *et al.*, 2013); *WRKY*s, e.g. *OsWRKY53* (Chujo *et al.*, 2007); and respiratory burst oxidase homologs, e.g. *OsrbohA* and *OsrbohE* (Yoshie *et al.*, 2005). A variety of genes involved in effector-triggered immunity (ETI) were also significantly up-regulated in the *spl33* mutant, including resistance (R) proteins, e.g. *RPM1* and *RPS2* (Mackey *et al.*, 2002, 2003); heat shock proteins, e.g. *HSP90* (Shirasu, 2009); suppressors of the G2 allele of skp1, e.g. *SGT1* (Shirasu, 2009); and *PR*s, e.g. *OsPR1a* and *OsPR1b* (Agrawal *et al.*, 2001). Collectively, both the PTI- and ETI-related receptor genes and signaling molecules were significantly up-regulated in *spl33*, suggesting that the loss-of-function of *SPL33* leads to negative regulation of innate immunity, including both PTI and ETI, reinforcement of cell walls, induction of anti-microbial proteins and accumulation of phytoalexins, thus resulting in broad-spectrum resistance of *spl33* to pathogens.

No previous report has suggested that an eEF1A-like protein functions in cell death and defense responses in plants. The present study identified a novel rice LMM caused by functional loss of an eEF1A-like protein. We characterized the roles of this eEF1A-like protein, and confirmed that lost function of eEF1A-like protein leads to cell death and induces defense responses in rice.

## Supplementary Material

Supplementary Data
