# Supplementary material for: SPL33, encoding an eEF1A-like protein, negatively regulates cell death and defense responses in rice
Source: J Exp Bot. 2017 Feb 11;68(5):899–913. doi: 10.1093/jxb/erx001 (PMC5441852; doi:10.1093/jxb/erx001)
Supplement: Supplementary Data [file erx001_Supplementary_Data.zip › Supplementary_figures_S1_S10.pdf]

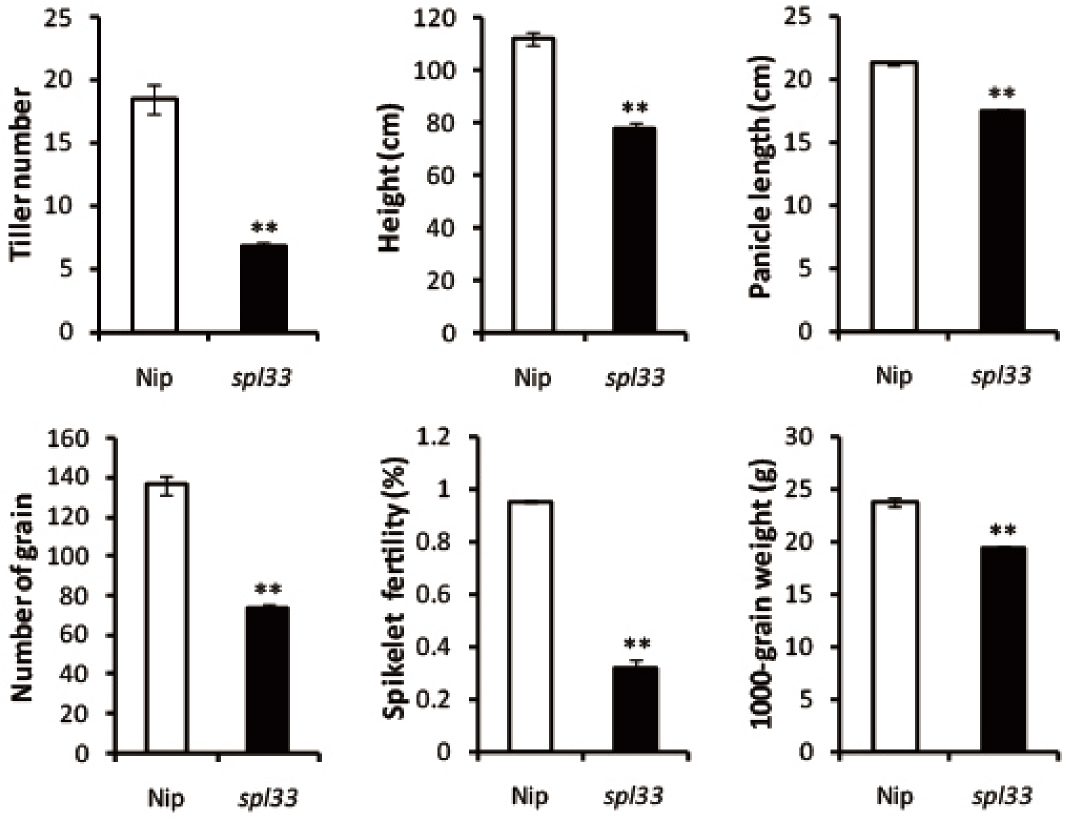

Supplementary Figure S1. Trait measurements of wild type Nipponbare (Nip) and *spl33*. Data are means ±SD of 12 plants (Student's *t*-test, **, $P < 0.01$).

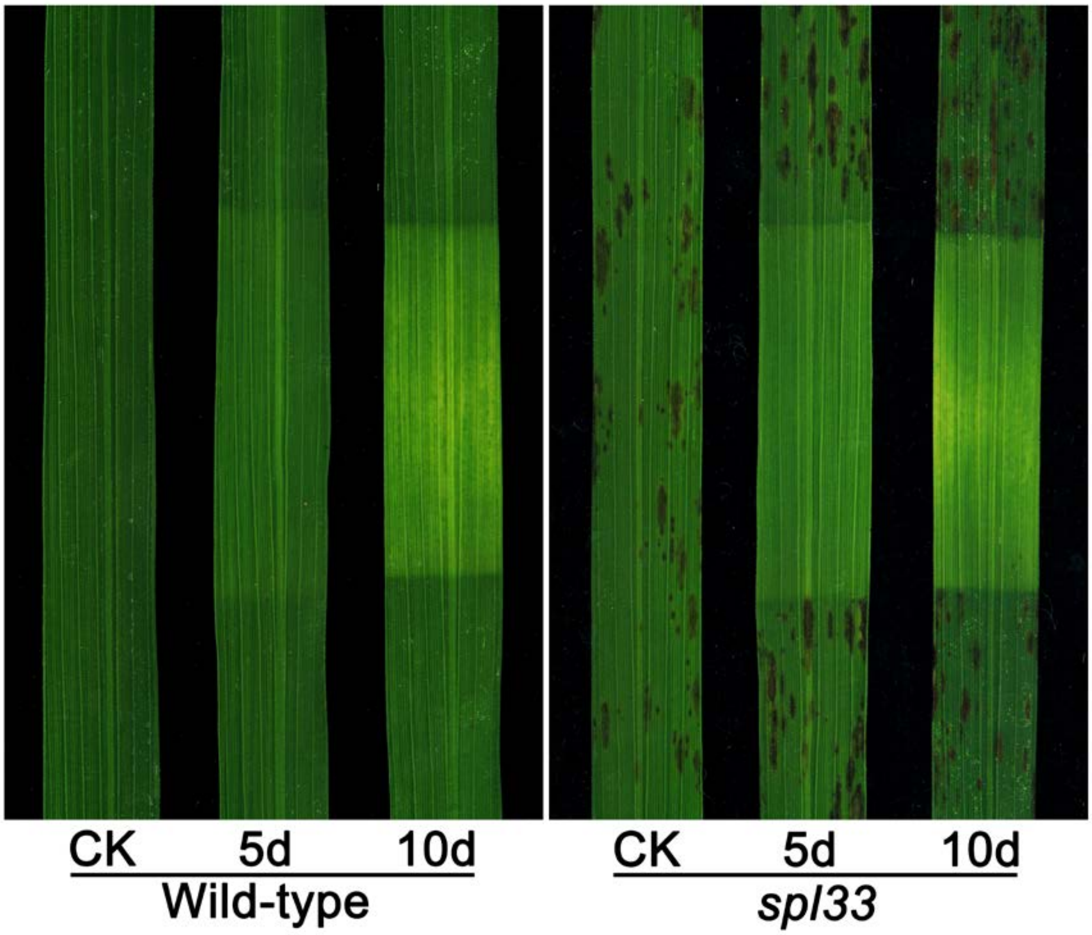

Supplementary Figure S2. Light-dependent development of lesions in *spl33*. From left to right: un-treated leaf blade; leaf blade blocked from light for 5 d; leaf blade blocked from light for 10 d.

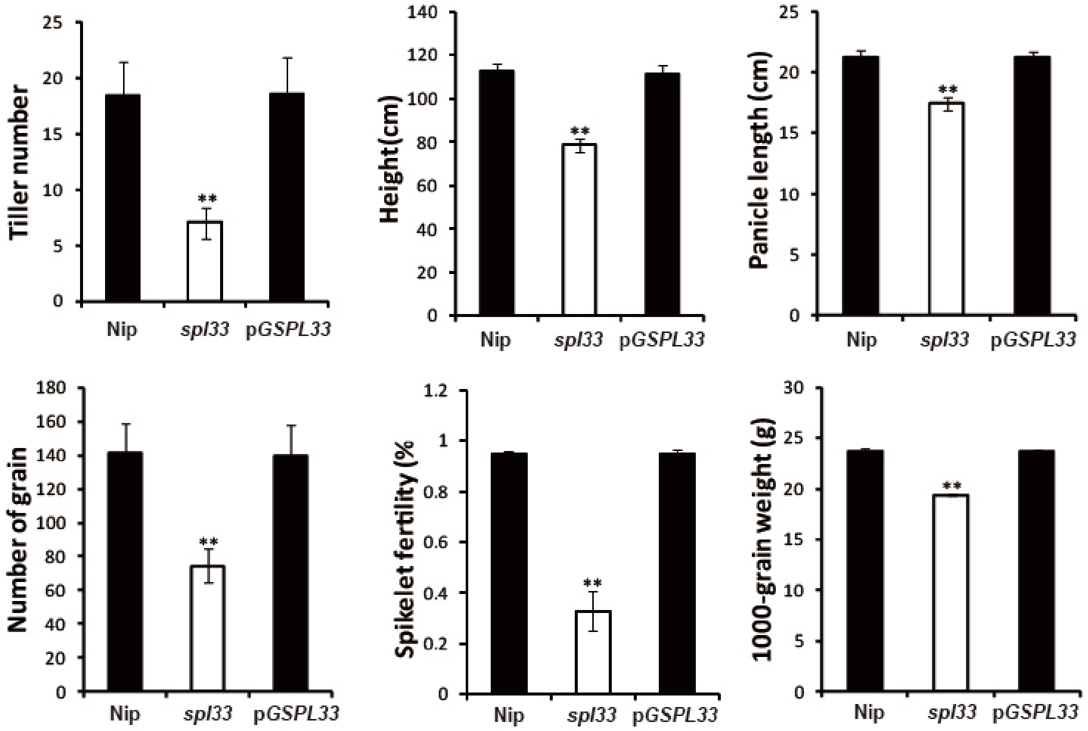

Supplementary Figure S3. Trait measurements of WT, *spl33* and T_1 complementary plants. Data are means ±SD of 12 plants (Student's *t*-test, **, $P < 0.01$).

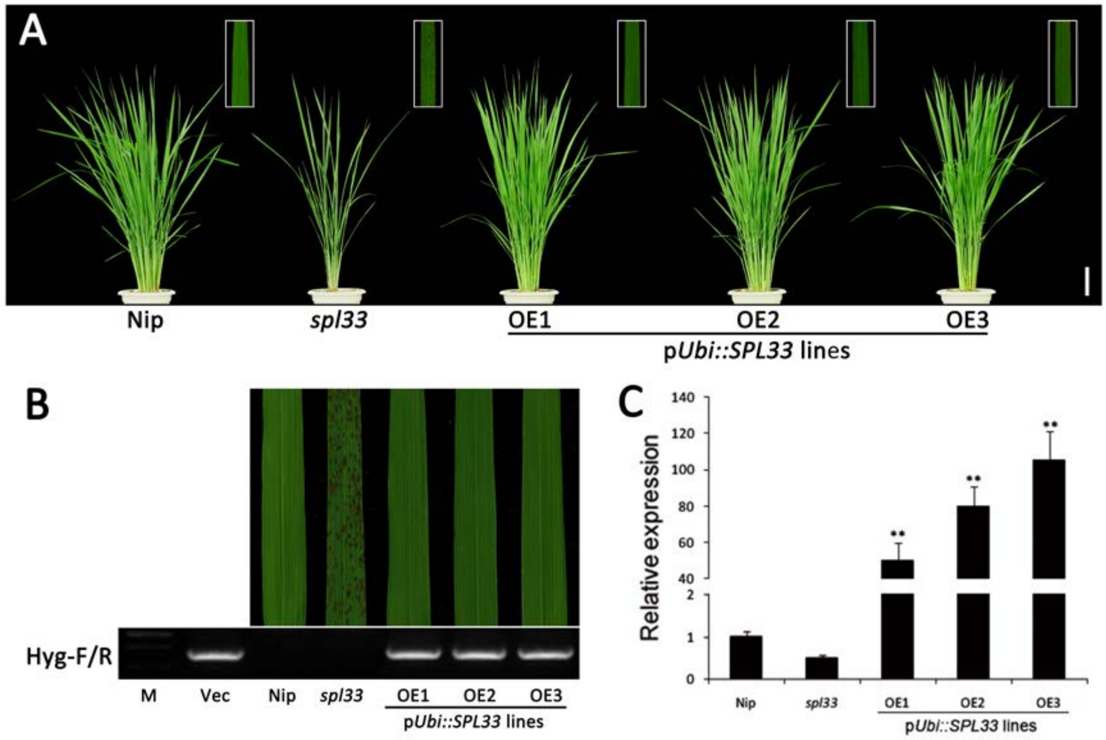

Supplementary Figure S4. The *SPL33* overexpression test. A. Phenotypes of wild type Nipponbare (Nip), *spl33* mutant, and 3 independent T_0 overexpression lines of p*Ubi::SPL33*. The insert indicates enlargement of leaf section with lesion spots. Bar, 10cm. B. Characterization of transgenic plants by PCR. M: molecular markers; Vec: the vector p*Ubi::SPL33*. C. qRT-PCR analysis in flag leaf of wild type (Nip), *spl33* mutant and T_0 overexpression lines. The expression level of each gene in WT was normalized to 1. Data represent means ±SD of three biological replicates (Student's *t*-test, **, $P < 0.01$).

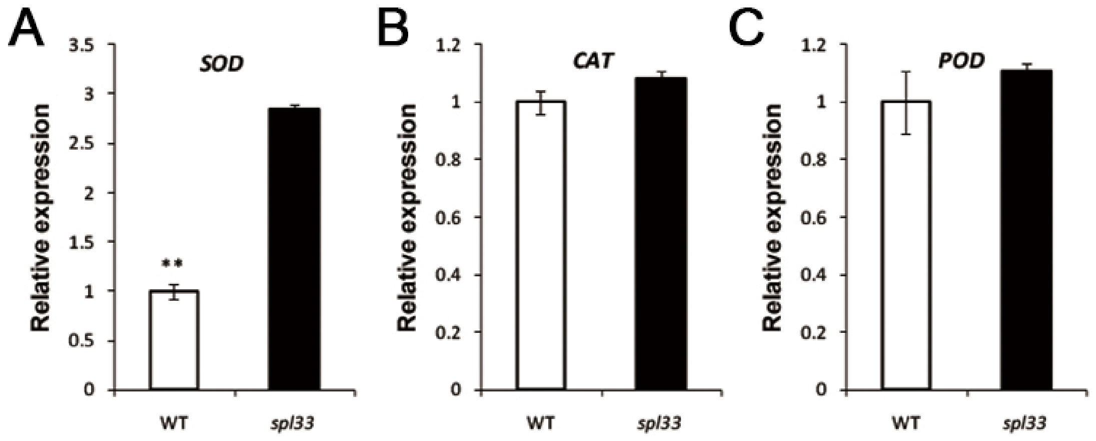

Supplementary Figure S5. Transcription analysis of anti-oxidative enzymes in WT and *spl33* leaves. RNA was extracted from leaves of the 28-day-old *spl33* and WT plants. The expression level of each gene in WT was normalized to 1. Data represent means ±SD of three biological replicates (Student's *t*-test, **, $P < 0.01$). SOD, superoxide dismutase; CAT, catalase; and POD, peroxidase.

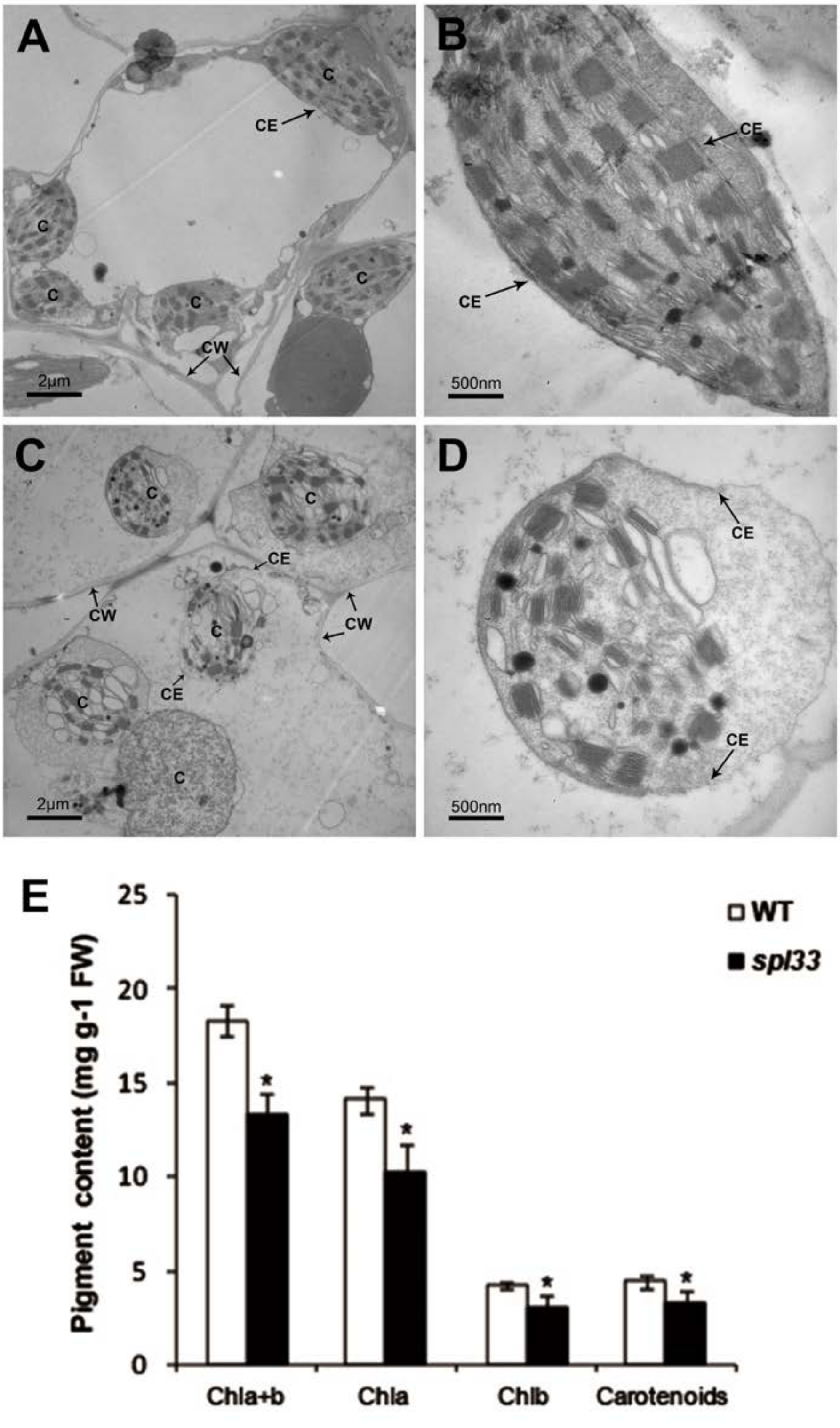

Supplementary Figure S6. Ultrastructure of chloroplasts in mesophyll cells of WT and *spl33*. A-D. Electron micrographs of chloroplasts in WT (A-B) showing chloroplasts with well-ordered thylakoid and membrane stacking in mesophyll cells, and in *spl33* (C-D) showing degraded chloroplasts. E. Pigment contents in leaves of WT and *spl33* mutant in mg/g fresh weight (FW). Data are means ±SD of three biological replicates (Student's *t*-test: *, $P < 0.05$). C, chloroplast; CW, cell wall; CE, chloroplast envelope; Chla, Chlorophyll a; Chlb, chlorophyll b. Bar = 1 μm in A and C and 500 nm in B and D.

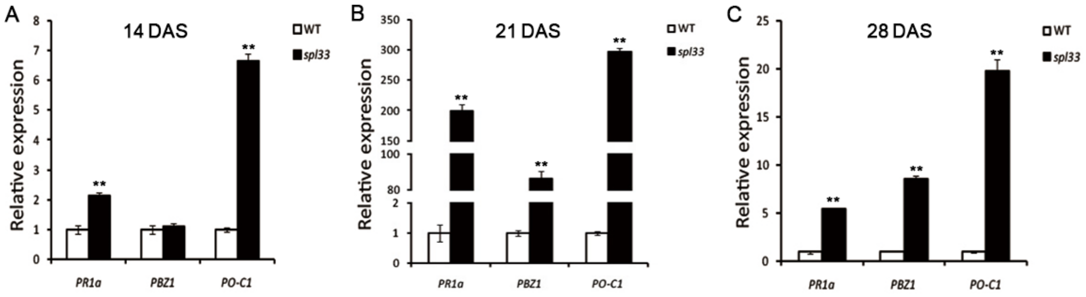

Supplementary Figure S7. Expression analysis of three defense marker genes in leaves of WT and *spl33* seedlings. RNA was extracted from leaves of the 14- (A), 21- (B) and 28-day-old (C) *spl33* and WT plants. Expression level of each gene in wild type was normalized to 1. Data are means ±SD of three biological replicates (Student's *t*-test: **, $P < 0.01$).

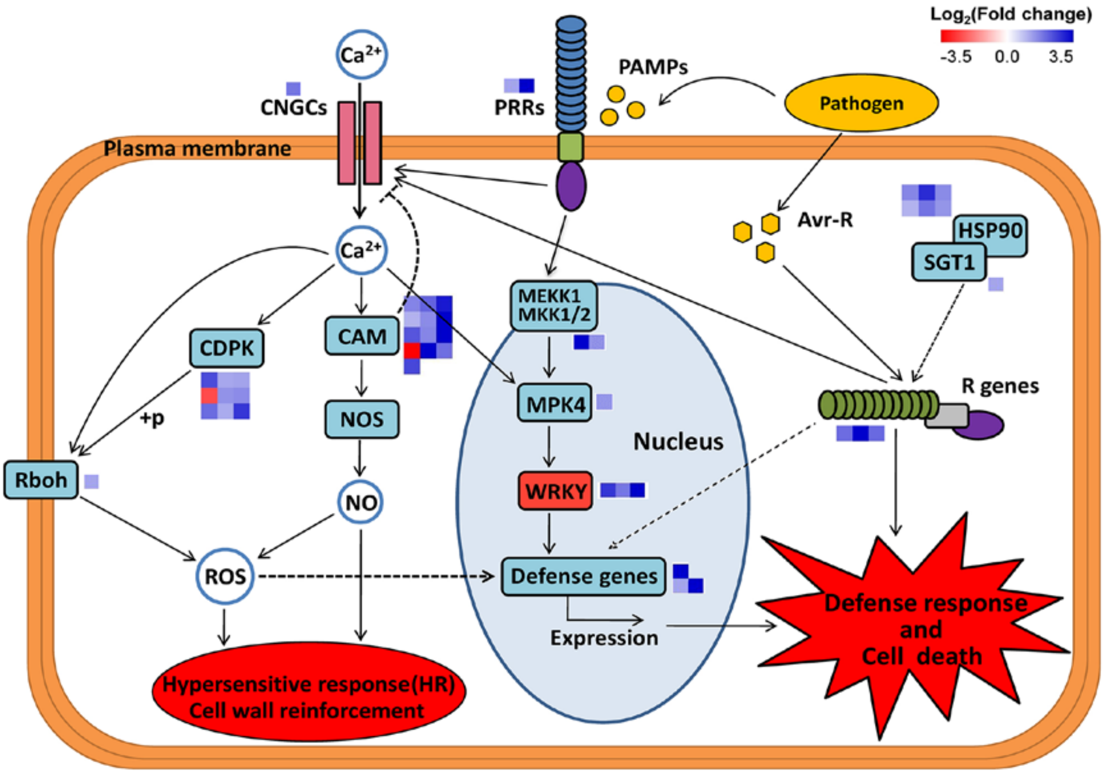

Supplementary Figure S8. Overview of differentially regulated genes involved in plant-pathogen interaction pathway. Genes induced or repressed by *spl33* mutation are shown in blue and red colors, respectively, as shown in the color bar ranging from -3.5 to +3.5 (log_2 fold change). One block represents one gene. PAMPs, pathogen-associated molecular patterns; PRRs, pattern recognition receptors; Avr-R, avirulence genes; R genes, resistance genes; *HSP90*, heat shock protein 90; *SGT1*, suppessor of the G2 allele of skp1; *MEKK1*, MEK kinase 1; *MKKs*, MAP kinase kinases; *WRKY*, transcription factor; *CNGCs*, cyclic nucleotide-gated channels; *CAM*, calmodulin-like protein; *CDPK*, Calcium-Dependent Protein Kinase; *Rboh*, rice respiratory burst oxidase homolog; *NOS*, nitric oxide synthase.

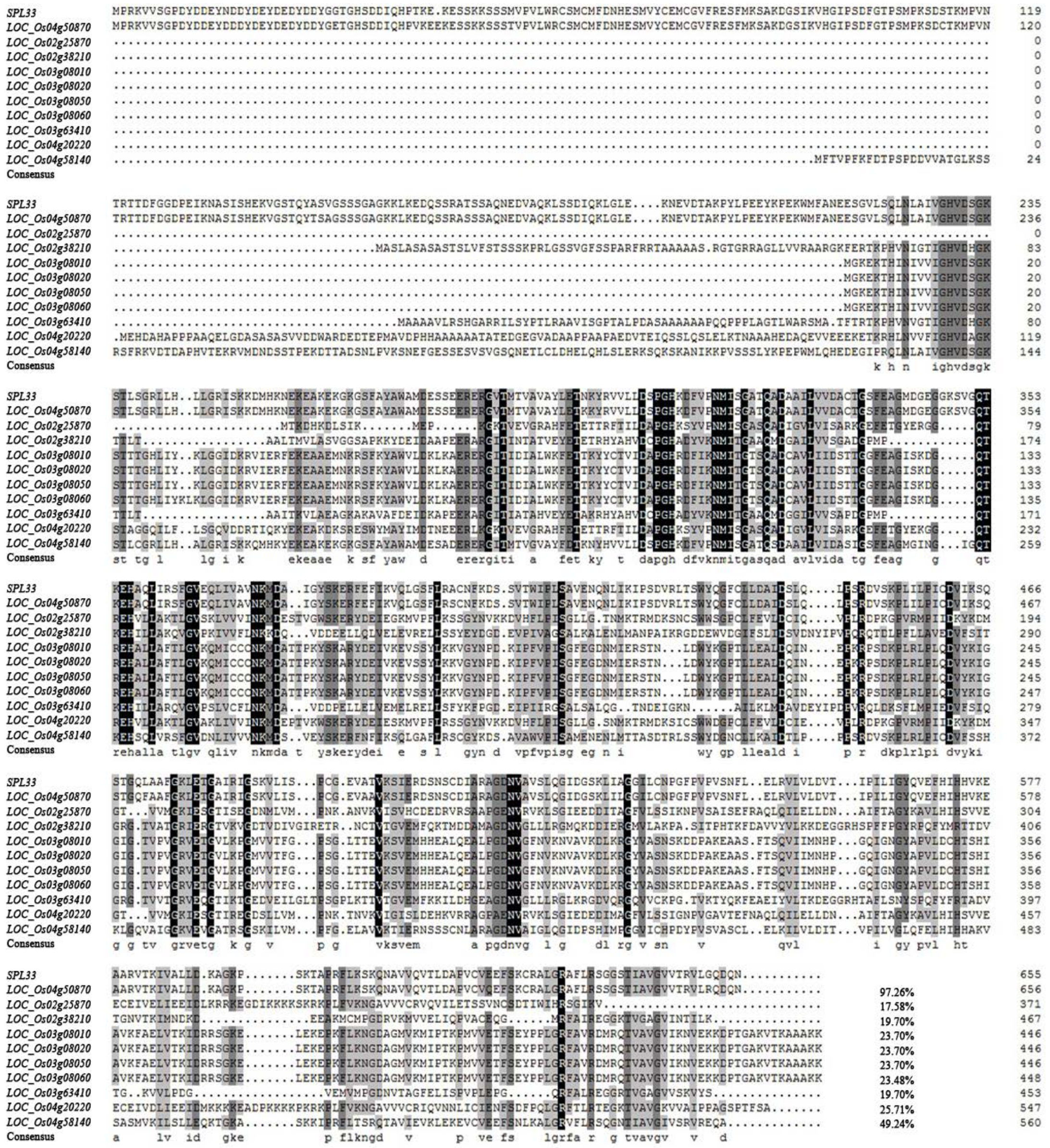

Supplementary Figure S9. Comparison of amino acid sequences of 11 putative eEF1A proteins in rice.

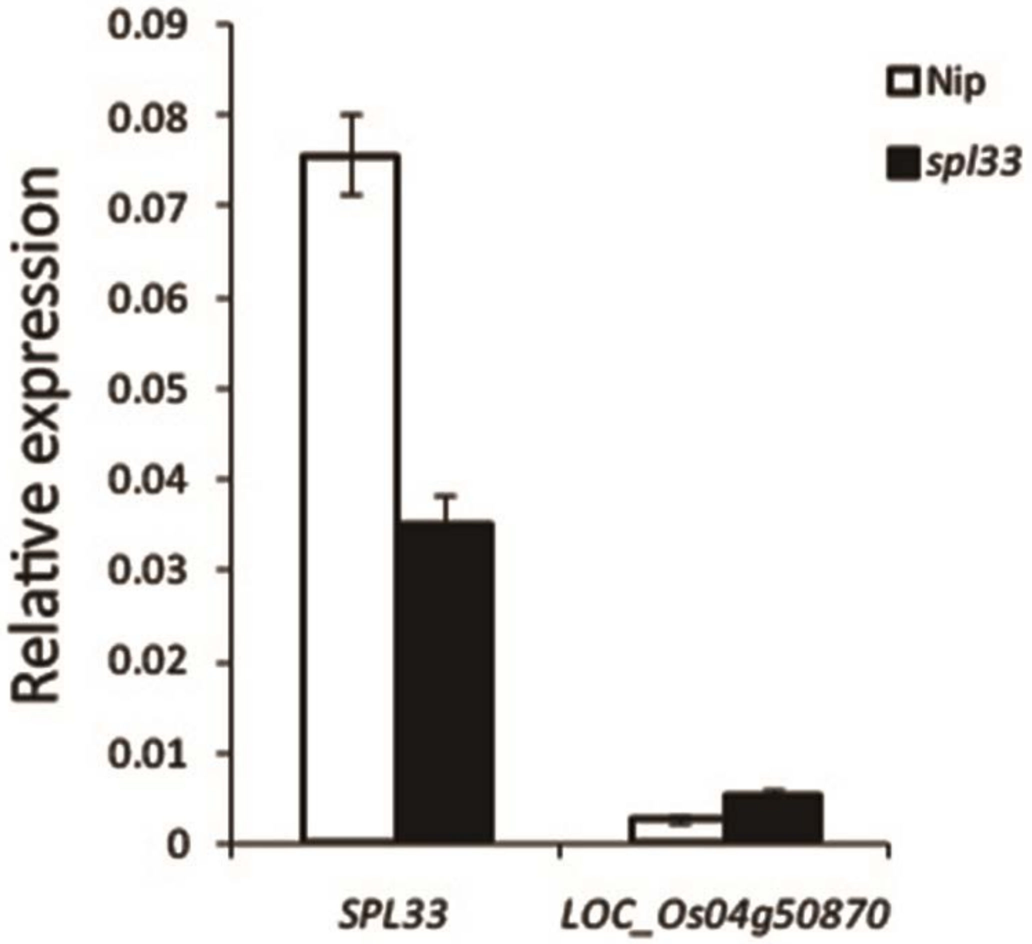

Supplementary Figure S10. Expression analysis of *SPL33* and *LOC_Os04g50870* in leaves of WT and *spl33* seedlings. RNA was extracted from leaves of the 28-day-old *spl33* and WT plants. Data represent means ±SD of three biological replicates.
