# Supplementary material for: SPL33, encoding an eEF1A-like protein, negatively regulates cell death and defense responses in rice
Source: J Exp Bot. 2017 Feb 11;68(5):899–913. doi: 10.1093/jxb/erx001 (PMC5441852; doi:10.1093/jxb/erx001)
Supplement: Supplementary Data [file erx001_Supplementary_Data.zip › supplementary_tables_S1_S6.pdf]

Supplementary Table S1. InDel markers used in this study

Marker	Primer sequences（5′ → 3′）	
	Forward primer	Reverse primer
I1-1	AGGGGAAGAAAAACCTGACC	CCGCGTGCAGATAAAGTACA
S1-1-5	GCTGCTCGAACCCTAGATTG	ATTTCCCAGTTCCCACATCA
L4	AGCGTGGACCCAAAGTAGTG	TCAGCTCCAAATACCCTTGG
L31	GGGGATGAAGGGTAATTTCG	CAGCTGCAAGACCCATTTTT
L70	CACGTCGTGGAGGACTTCTT	GAATACGCGCACCCTGAG
V10	TTACGGTGGAACCAAACACA	TTTTGGACGAATGTGATGCT
V16	AAGGAGAACCGGAAAGCAAC	TGTCCATGCCATAACAATGC
V19	TGGCAAAATTAGCAAAACCA	GGCGACAATTCTTGCTTACC
V34	ACGGCTAGTTCCGCTACGTT	CACAGTCCACACCACGTCAC
V43	CTGCCTGCTGTTGTTGTGTT	CCACCGGCTTCACAATTC
V54	CCAGCAGTCCACTTCAAAATC	ACAGGGAGCAAGGAAAGGAT
H2	TCATGGCCAAGTATCCACAA	TTACTTGGGTCGGCTTTGTC
H23	AAGGAAGCACGTTCAAGCAT	CCTCACGCATCATTATATGTCC

Supplementary Table S2. Primers used for vector construction

Marker	Vector	Primer sequences（5′ → 3′）	
		Forward primer	Reverse primer
pP1305	pCAMBIA 1305.2	CCATGATTACGAATTCTCT	CTCAGATCTACCATGGCAC
		CAACGAGTTTTATGGCT	AGGGCAGACCTAATGGG
pC1390	pCUbi1390	GCAGGTCGACGGATCCCCACC	GAATTCCCGGGGATCCGGCCA
		GAGCCACTTCATACTTCAAT	TGTCGTAATTTCCTCCCTAT
Pro-SPL33	pCAMBIA 1305.1	CGGTACCCGGGGATCCTCT	ATTTACCCTCAGATCTCAC
		CAACGAGTTTTATGGCT	AGGGCAGACCTAATGGG
SPL33-GFP	pAN580	GCCCAGATCAACTAGTATGC	TCGAGACGTCTCTAGAGTTC
		CGCGCAAGGTTGTCTCTGGG	TGATCTTGCCCGAGTACCCG
1305GFP-SPL33	pCAMBIA 1305.1-GFP	CGGTCCCGGGGGATCCCCAC	TGCTCACCATGGATCCGTTC
		CGAGCCACTTCATACTTCAAT	TGATCTTGCCCGAGTACCCG
p1390-SPL33$^{1-223}$	pCUbi1390	GCAGGTCGACGGATCCATGCCG	GAATTCCCGGGGATCCTCAT
		CGCAAGGTTGTCTCTGGGC	AGTTGGCTCAGCACTCCTGAT
p1390-SPL33$^{224-655}$	pCUbi1390	GCAGGTCGACGGATCCATGA	GAATTCCCGGGGATCCTCAGT
		ACCTTGCAATAGTAGGTCATG	TCTGATCTTGCCCGAGTACC

Supplementary Table S3. Primers used for quantitative real-time PCR

Marker	Primer sequences（5′ → 3′）	
	Forward primer	Reverse primer
Ubi	ACCCTGGCTGACTACAACATC	AGTTGACAGCCCTAGGGTG
SPL33	TGTGGCTTATTTGGAGACCA	TGCAAGCATCAACAACAAGA
SOD	TCCGCCGTATAAACTTGATGCCCT	TGGGTTGCCGTTGTTGTATGCTTC
CAT	GCTTGCTTTCTGCCCAGCGATAAT	AAATAGTTTGGGCCAAGACGGTGC
POD	AACCCATCCTACGCCAGA	CGCCTTGAGGTTCACGAAGTA
porA	ATCACCAAGGGCTACGTCTC	GAGTTGTTGTTCCAGCTCCA
rbcL	GTTGAAAGGGATAAGTTGA	AATGGTTGTGAGTTTACG
rbcS	TCATCAGCTTCATCGCCTAC	ACTGGGAACACACGAAACAA
psaA	GAGATACCACTTCCTCAT	ACTAAGAAATTCTGCGTATT
psaB	TTGGTATTGCTACCGCACAT	CCGGACGTCCATAGAAAGAT
psbA	AAGTTTCTCTGATGGTATG	ATAGCACTGAATAGGGAA
psbB	TCATATTGCTGCGGGTACAT	AGTTGCTGACCCATACCACA
psbC	TACAACCTTGGCAAGAACGA	TACGCCACCCACAGAATTTA
cab2R	GTTCTCCATGTTCGGCTTCT	GACGAAGTTGGTGGCGTAG
rpoA	TCAGGGAATTCACCATGCTA	ATCAAATTGGTCAGGGTGGT
CHLI	AGTAACCTTGGTGCTGTG	AATCCATCAACATTCAACTCTG
CHLD	GGAAAGAGAGGGCATTAG	CAATACGATCAAGTAAGTGTT
SGR	GGCCTCCGCTACTACATCTT	TTGGAGTGGAAGTAGACCCA
Osl2	GCAGACAACAAATCGCCAAAT	TCTCCAGCAACTCTAACCAGCAT
Osl30	GAGAAATCCCTTGAAGCCAA	CACAAAGCAGTGAAAGCACA
Osl43	ATTCAGCATTCCACTGCAAG	ATTCAGCATTCCACTGCAAG
Osl57	ATTACAGGCAGTGGCTGATG	ATTACAGGCAGTGGCTGATG
Osh36	GTGCACCATGCACTTAATCC	CACCGACCCTTCCTGTAGTT
Osh69	ACGAGCTACACGCCTACCTT	ACTTCCTTGCCAGAAGCACT
PR1a	TTCATCACCTGCAACTACTCG	TGCATAAACACGTAGCATAGCAT
PBZ1	GGTGTGGGAAGCACATACAA	GTCTCCGTCGAGTGTGACTTG
PO-C1	GCTCTGTTTCTTGTTCCATCTG	AGTATAATCCTCCGTGCCTTTG
LOC_Os04g 50870	TTGAACGTATCTGCAGCCGT	TACAGGCCATATCTCAACGC

Supplementary Table S4. The segregation analysis of lesion phenotype in F_2 populations derived from crosses of *spl33*/WT or WT/*spl33*

Cross	No. of F_2 individuals			$\chi^2_{0.05}$ <3.841
	Normal	Lesion mimics	Total	
spl33 × Nip	874	274	1148	0.785
Nip × *spl33*	745	243	988	0.086

Supplementary Table S5. Eleven open reading frames in the target 70 kb region

Gene	Gene ID	Physical locus	Description
ORF1	*LOC_Os01g02690*	915639-919674	Resistance-related receptor-like kinase, putative, expressed
ORF2	*LOC_Os01g02700*	920992-923630	Protein kinase domain containing protein, expressed
ORF3	*LOC_Os01g02710*	924451- 925175	LRk-type protein, putative, expressed
ORF4	*LOC_Os01g02720*	929883-934914	Elongation factor Tu, putative, expressed
ORF5	*LOC_Os01g02730*	935574-938367	TAK14, putative, expressed
ORF6	*LOC_Os01g02740*	940005-952451	Retrotransposon protein, putative, unclassified, expressed
ORF7	*LOC_Os01g02750*	954075-956817	Protein kinase domain containing protein, expressed
ORF8	*LOC_Os01g02760*	957907-960626	Receptor-like kinase, putative, expressed
ORF9	*LOC_Os01g02770*	961681-965151	Resistance-related receptor-like kinase, putative, expressed
ORF10	*LOC_Os01g02780*	969308-973327	TAK33, putative, expressed
ORF11	*LOC_Os01g02790*	976766-980431	Protein kinase domain containing protein, expressed

Supplementary Table S6. SA and JA/ETH biosynthesis-associated genes in DEGs

Gene ID	Rice symbol	Arabidopsis symbol	Description	WT value	*spl33* value	log2FC	FDR
Information of eight SA biosynthesis-associated genes detected among 4,792 DEGs							
LOC_Os02g41650	*OsPAL2*	*PAL1*	phenylalanine ammonia-lyase, putative, expressed	76.17	168.6	1.15	8.49E-05
LOC_Os02g41670	*OsPAL3*	*PAL1*	phenylalanine ammonia-lyase, putative, expressed	0.02	11.97	9.19	0.005468
LOC_Os02g41680	*OsPAL4*	*PAL1*	phenylalanine ammonia-lyase, putative, expressed	1.77	105.23	5.89	8.49E-05
LOC_Os04g43760	*OsPAL5*	*PAL1*	phenylalanine ammonia-lyase, putative, expressed	22.07	8.87	-1.31	8.49E-05
LOC_Os04g43800	*OsPAL6*	*PAL1*	phenylalanine ammonia-lyase, putative, expressed	46.73	158.32	1.76	8.49E-05
LOC_Os05g35290	*OsPAL7*	*PAL1*	phenylalanine ammonia-lyase, putative, expressed	5.59	26.87	2.26	8.49E-05
LOC_Os11g48110	*OsPAL8*	*PAL1*	phenylalanine ammonia-lyase, putative, expressed	1.249	3.82	1.61	8.49E-05
LOC_Os12g33610	*OsPAL9*	*PAL1*	phenylalanine ammonia-lyase, putative, expressed	57.13	150.42	1.39	8.49E-05
Information of 21 JA biosynthesis-associated genes detected among 4,792 DEGs							
LOC_Os03g49380	*OsLOX5*	*LOX5*	lipoxygenase, putative, expressed	2.89	7.07	1.29	8.49E-05
LOC_Os08g39840	*OsLOX9*	*LOX2*	lipoxygenase, chloroplast precursor, putative, expressed	0.15	142.78	9.89	8.49E-05
LOC_Os08g39850	*OsLOX8*	*LOX2*	lipoxygenase, chloroplast precursor, putative, expressed	0.50	172.53	8.42	8.49E-05
LOC_Os12g37320	—	*LOX2*	lipoxygenase 2.2, chloroplast precursor, putative, expressed	0.15	9.62	5.94	8.49E-05
LOC_Os12g37260	*OsLOX11*	*LOX2*	lipoxygenase 2.1, chloroplast precursor, putative, expressed	2018.61	575040.1	8.16	0
LOC_Os03g52860	*L-2*	*LOX5*	lipoxygenase, putative, expressed	5.74	165.47	6.32	2.11E-12
LOC_Os03g49350	*OsLOX4*	*LOX5*	lipoxygenase protein, putative, expressed	53.97	120.18	1.1	2.32E-16
LOC_Os03g12500	*OsAOS2*	*AOS*	cytochrome P450, putative, expressed	0.34	297.39	9.78	8.49E-05
LOC_Os03g55800	*OsAOS1*	*AOS*	cytochrome P450, putative, expressed	5.73	17.15	1.58	8.49E-05
LOC_Os01g27230	—	*OPR2*	12-oxophytodienoate reductase, putative, expressed	11.36	4.86	-1.22	8.49E-05
LOC_Os01g27240	—	*OPR2*	12-oxophytodienoate reductase, putative, expressed	0.37	0.91	1.29	0.000166
LOC_Os06g11240	—	*OPR2*	12-oxophytodienoate reductase, putative, expressed	0.33	32.05	6.59	8.49E-05
LOC_Os06g11280	—	*OPR2*	12-oxophytodienoate reductase, putative, expressed	0.98	0.3	-1.68	0.000166
LOC_Os06g11290	*OsOPR1*	*OPR2*	12-oxophytodienoate reductase, putative, expressed	0.77	3.81	2.31	8.49E-05
LOC_Os08g35740	*OsOPR7*	*OPR3*	12-oxophytodienoate reductase, putative, expressed	26.96	61.11	1.18	8.49E-05
LOC_Os06g11210	—	*OPR1*	12-oxophytodienoate reductase, putative, expressed	0	402.35	11.65	0
LOC_Os05g07090	—	*ACX4*	acyl-coenzyme A dehydrogenase, mitochondrial precursor, putative, expressed	6.74	23.19	1.78	8.49E-05
LOC_Os06g24704	*OsACX3*	*ACX3*	acyl-coenzyme A oxidase, putative, expressed	2.87	8.43	1.56	8.49E-05
LOC_Os06g23780	—	*ACX4*	glutaryl-CoA dehydrogenase, mitochondrial	1.15	300.46	7.84	4.49E-23

			precursor, putative, expressed				
LOC_Os06g23760	—	*ACX4*	acyl-CoA oxidase, putative, expressed	0	66.19	8.98	1.71E-51
LOC_Os10g31950	—	*KAT2*	3-ketoacyl-CoA thiolase, peroxisomal precursor, putative, expressed	8.46922	33.08	1.97	8.49E-05
Information of three ETH biosynthesis-associated genes detected among 4,792 DEGs							
LOC_Os01g39860	*OsACO7*	*ACO1*	lipoxygenase, putative, expressed	0.14	12.19	6.42	8.49E-05
LOC_Os09g27820	*OsACO1*	*ACO4*	lipoxygenase, chloroplast precursor, putative, expressed	0.26	1.41	2.42	8.49E-05
LOC_Os04g48850	*OsACS2*	*ACS6*	aminotransferase, classes I and II, domain containing protein, expressed	0	1448.29	13.53	0
